# Precision mitochondrial DNA editing with high-fidelity DddA-derived base editors

**DOI:** 10.1038/s41587-022-01486-w

**Published:** 2022-10-13

**Authors:** Seonghyun Lee, Hyunji Lee, Gayoung Baek, Jin-Soo Kim

**Affiliations:** 1 Center for Genome Engineering, Institute for Basic Science, Daejeon, Republic of Korea; 2 Laboratory Animal Resource Center, Korea Research Institute of Bioscience and Biotechnology, Daejeon, Republic of Korea

**Keywords:** Genetic engineering, Protein design

## Abstract

Bacterial toxin DddA-derived cytosine base editors (DdCBEs)—composed of split DddA_tox_ (a cytosine deaminase specific to double-stranded DNA), custom-designed TALE (transcription activator-like effector) DNA-binding proteins, and a uracil glycosylase inhibitor—enable mitochondrial DNA (mtDNA) editing in human cells, which may pave the way for therapeutic correction of pathogenic mtDNA mutations in patients. The utility of DdCBEs has been limited by off-target activity, which is probably caused by spontaneous assembly of the split DddA_tox_ deaminase enzyme, independent of DNA-binding interactions. We engineered high-fidelity DddA-derived cytosine base editors (HiFi-DdCBEs) with minimal off-target activity by substituting alanine for amino acid residues at the interface between the split DddA_tox_ halves. The resulting domains cannot form a functional deaminase without binding of their linked TALE proteins at adjacent sites on DNA. Whole mitochondrial genome sequencing shows that, unlike conventional DdCBEs, which induce hundreds of unwanted off-target C-to-T conversions in human mtDNA, HiFi-DdCBEs are highly efficient and precise, avoiding collateral off-target mutations, and as such, they will probably be desirable for therapeutic applications.

## Abstract (web summary)

Off-target activity of DddA-derived base editors is minimized through protein engineering.

## Main

DdCBEs^1^ and zinc finger deaminases (ZFDs)^2^—composed of the split interbacterial toxin DddA_tox_, a uracil glycosylase inhibitor (UGI), and custom-designed DNA-binding TALE arrays and zinc finger proteins, respectively—are highly versatile genome editing tools that enable targeted C-to-T conversions in the nuclear genome and mitochondrial^2–9^ and chloroplast DNA^10–12^. DddA_tox_, an enzymatic moiety in the interbacterial toxin DddA derived from *Burkholderia cenocepacia*, catalyzes cytosine deamination within double-strand DNA (dsDNA)^13^. To avoid toxicity in host cells, DddA_tox_ is split into two inactive halves, each of which is fused to custom-designed TALE DNA-binding proteins to make a functional DdCBE pair. In principle, the deaminase enzyme activity is reconstituted only when the two inactive halves are brought together on target DNA by two adjacently bound TALE proteins. C-to-T base conversions are induced between the two TALE-binding sites in a spacer region.

**Fig. 1:**
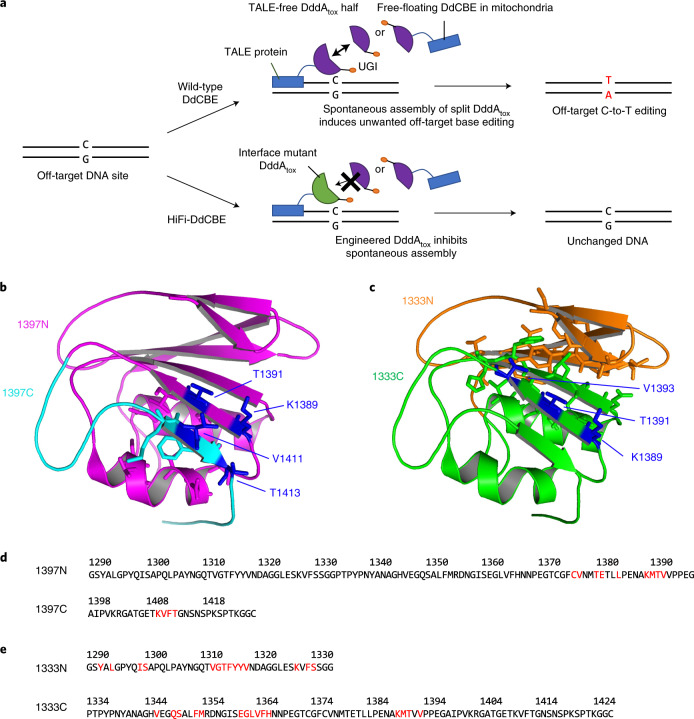
Interface engineering of two split DddA_tox_ proteins. **a**, Schematic of DdCBE engineering. **b**,**c**, Crystal structures of the DddA_tox_ deaminases. Residues at the interfaces of the split dimers are represented as sticks. In **b**, 1397N and 1397C are shown in magenta and cyan, respectively. In **c**, 1333N and 1333C are shown in orange and green, respectively. **d**,**e**, Interface mutations in 1397N and 1397C (**d**) and in 1333N and 1333C (**e**) are shown in red.

Recently, it has been shown that DdCBEs targeted to mitochondrial genes in human cells^14^ and mice^5,15^ and to chloroplast genes in plants^10,11^ can cause off-target mutations, raising concerns about the specificity of DdCBEs. We reasoned that DdCBE off-target activity might arise from two sources: nonspecific TALE–DNA interactions; and spontaneous assembly of split DddA_tox_ halves independent of TALE–DNA interactions (Fig. 1a). The former depends on TALE proteins and the choice of target sequences (some sites may have many similar sites in the genome), whereas the latter is a more general problem. In this study, we focused on split DddA_tox_ halves and sought to engineer the split dimer interface (Fig. 1b–e) to avoid spontaneous assembly, an approach that has been successfully used to improve the specificity of dimeric zinc finger nucleases^16,17^.

## Results

### Off-target base editing with a TALE-free DddA split half

**Fig. 2:**
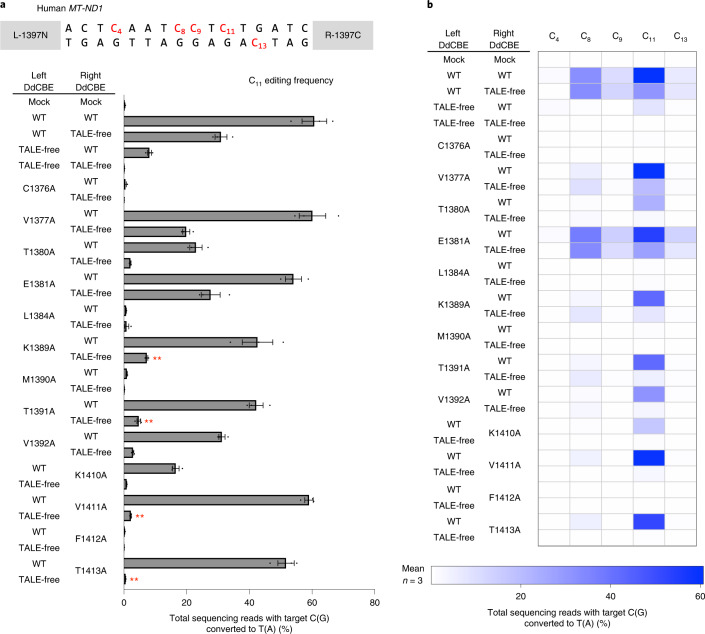
Cytosine base editing induced by G1397-split DdCBE and various mutants in human mtDNA. **a**, Base editing frequencies induced by G1397-split DddA_tox_ interface mutants. The editing window and target cytosine bases are shown at the top. Plasmids encoding the mutant and wild-type (WT) or TALE-free DddA_tox_ proteins were co-transfected as indicated in the left column. All of the TALE-fused and TALE-free constructs contain UGI. **b**, Heat map showing target C-to-T (G-to-A) editing efficiencies induced by DdCBE and the various mutants. Error bars are s.e.m. for *n* = 3 biologically independent samples. *P* = 0.0045, *P* = 0.0032, *P* = 0.0070 and *P* = 0.0066 for K1389A, T1391A, V1411A and T1413A, compared to wild-type and TALE-free pair, respectively. **P < 0.01, Student’s two-tailed *t*-test. Source data

We investigated whether one DdCBE subunit alone bound to a target site in the human mitochondrial gene could catalyze C-to-T conversions when paired with a TALE-free DddA_tox_ half with no apparent target site in the mtDNA. To test this hypothesis, we chose a DdCBE pair (L-1397N (the amino-terminal DddA_tox_ half split at G1397 fused to the carboxyl terminus of the TALE array designed to bind to the left half-site) plus R-1397C (the C-terminal DddA_tox_ half split at G1397 fused to the C terminus of the TALE array designed to bind to the right half-site)) specific to the *MT-ND1* gene, which catalysed C-to-T conversions efficiently with a frequency of 60.7% ± 3.2% at position 11 (C_11_) at the target site in HEK293T cells (Fig. 2a). Surprisingly, each subunit alone (L-1397N or R-1397C), paired with the other TALE-free DddA_tox_ half, also induced base editing, albeit less efficiently than did the original pair. Thus, L-1397N alone and R-1397C alone bound to the *ND1* target site showed base editing with a frequency of 31% ± 1.6% and 8.1% ± 0.5%, respectively (Fig. 2). In other words, the DdCBE pair with two TALE fusions was more efficient than unmatched pairs with only one TALE fusion by merely 2.0-fold (60.7%/31%) or 7.5-fold (60.7%/8.1%). Apparently, the DddA_tox_ N-terminal moiety fused to a TALE array bound to a half-site can recruit the DddA_tox_ C-terminal moiety with no TALE array or vice versa to reconstitute a functional deaminase.

**Fig. 3:**
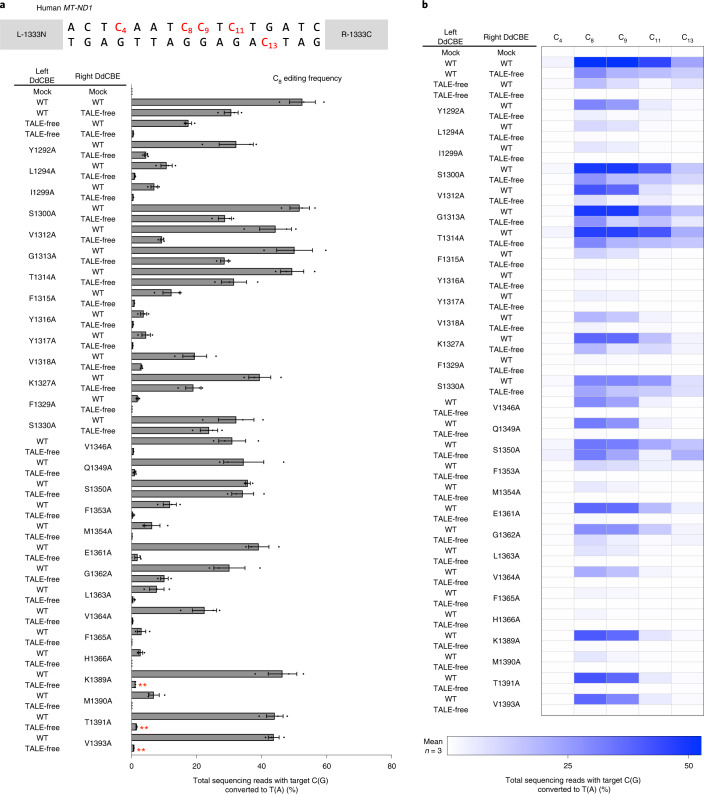
Target C-to-T conversions using G1333-split DdCBE and interface mutants in the *MT-ND1* gene. **a**, Base editing frequencies induced by G1333-split DddA_tox_ interface mutants. The editing window and target cytosine bases are shown at the top. Plasmids encoding the mutant and wild-type (WT) TALE-free DddA_tox_ proteins were co-transfected as indicated in the left column. The TALE-free DddA_tox_ proteins used for the left and right DdCBEs were 1333N and 1333C, respectively. **b**, Heat map showing target C-to-T (G-to-A) editing efficiencies induced by DdCBE and the various mutants. Error bars are s.e.m. for *n* = 3 biologically independent samples. *P* = 0.0036, *P* = 0.0042 and *P* = 0.0034 for K1389A, T1391A and V1393A, compared to the wild-type and TALE-free pair, respectively. **P < 0.01, Student’s two-tailed *t*-test. Source data

We also constructed the other DdCBE pair with DddA_tox_ split at G1333 (L-1333N plus R-1333C), rather than G1397, specific to the *MT-ND1* gene and tested whether L-1333N alone or R-1333C alone, each paired with the TALE-free DddA_tox_ half split at G1333 (1333C or 1333N, respectively), could induce C-to-T edits. Again, each TALE fusion alone showed base editing at C_8_, with a frequency of 30.7% ± 1.7% (L-1333N) or 17.5% ± 0.8% (R-1333C), whereas the original DdCBE pair induced base edits at a frequency of 52.5% ± 3.2% (Fig. 3a). Thus, the original pair with two TALE fusions was more efficient than the unmatched pairs with one TALE fusion by merely 1.7-fold (52.5%/30.7%) or 3.0-fold (52.5%/17.5%). Taken together, these results suggest that DdCBEs can cause unwanted off-target mutations at sites where only one TALE array can bind. Because TALE proteins can bind to sites with a few mismatches^18–20^, DdCBE pairs probably induce many off-target mutations in the organelle or nuclear genome.

### Split-dimer interface engineering of DddA_tox_

We sought to develop HiFi-DdCBEs that would not exhibit such off-target editing caused by spontaneous assembly of the split DddA_tox_ halves. We reasoned that the split dimer interface could be engineered to inhibit or prevent self-assembly. To this end, we first identified amino acid residues in the interface of the two split DddA_tox_ systems (split at G1333 and G1397). Because the crystal structure of DddA_tox_ bound to DNA was not available, we had to rely on the three-dimensional (3D) structure of DddA_tox_ complexed with DddI, the inhibitor protein (PDB ID: 6U08), in this analysis. We used a Python script in PyMOL (InterfaceResidues.py) to define the area of the surface of each split half and then to identify amino acid residues in each split half in close contact with those in the other split half. As a result, we chose nine amino acid residues in 1397N (the N-terminal DddA_tox_ half split at G1397), four residues in 1397C (the C-terminal DddA_tox_ half split at G1397), 14 residues in 1333Nand 15 residues in 1333C (Fig. 1b,c).

We then created a series of mutant DddA_tox_ halves in which each of these amino acid residues was replaced with alanine (Fig. 1d,e), an amino acid residue with a chemically inert and non-bulky side chain. Then we measured the editing frequencies of these interface mutant DdCBEs in combination with a wild-type DdCBE partner or a TALE-free DddA_tox_ half in HEK293T cells (Figs. 2 and 3). Many G1397-split DddA_tox_ variants, containing interface mutations such as C1376A, M1390A and F1412A, failed to induce C-to-T conversions in the spacer region between the two TALE-binding sites, even when combined with the wild-type partner, suggesting that these mutants cannot interact with other wild-type DddA_tox_ half nearby at the target site. Other DddA_tox_ variants, such as those containing V1377A and E1381A, induced C-to-T edits at high frequencies in partnership with the TALE-free half, comparable to the wild-type DdCBE pair, showing that these mutations are neutral and do not prevent split dimer interactions.

Variants containing several mutations such as K1389A, T1391A, V1411A and T1413A were highly active when combined with the wild-type DdCBE partner but poorly active with the TALE-free partner. For example, the V1411A variant in combination with the wild-type partner showed an editing efficiency of 59.0% ± 1.1%, on par with the wild-type DdCBE pair (60.7% ± 3.2%), but showed an efficiency of 2.2% ± 0.1% with the TALE-free construct, demonstrating a 26.9-fold difference in discrimination (59%/2.2%) at the C_11_ position. As indicated above, the wild-type G1333-split DdCBE pair showed a 7.5-fold difference in discrimination (60.7%/8.1%). Furthermore, these variants were more selective than the wild-type DdCBE pair. Thus, these variants edited C_11_ preferentially over C_8_, C_9_and C_13_ in the editing window, whereas the wild-type DdCBE pair was much less discriminatory, editing all four cytosines with high frequencies of >6.6% (Fig. 2b).

We also obtained several desirable interface mutations in the DdCBE pair with DddA_tox_ split at G1333 after screening 29 mutations (14 in 1333N and 15 in 1333C) (Fig. 3). Variants containing most of these mutations (such as I1299A, Y1316A, Y1317A and F1329A) were either poorly active even in combination with the wild-type partner or, in the case of other mutations (such as S1300A and T1314A), undesirably active in combination with the TALE-free partner. Notably, variants containing several mutations, including K1389A, T1391A and V1393A, were highly active when paired with the wild-type partner but inefficient when paired with the TALE-free partner. For example, the K1389A variant showed a 38.5-fold difference in discrimination (46.5% ± 3.6%/1.2% ± 0.02%), whereas the wild-type DdCBE pair showed a 3-fold difference in discrimination (52.5% ± 3.2%/17.5% ± 0.8%) at the C_8_ position. Furthermore, the pair containing the K1389A variant was more selective than the wild-type pair. Thus, this variant edited C_8_ preferentially over C_9_, C_11_ and C_13_, whereas the wild-type pair was promiscuous, editing all four cytosines with high frequencies of >19%. Note that C_11_ was preferentially edited by the pair containing V1411A in 1397C (Fig. 2b), whereas C_8_ was preferentially edited by the pair containing K1389A in 1333C (Fig. 3b). By contrast, both wild-type DdCBE pairs (DddA_tox_ split at G1333 or G1397) exhibited poor discrimination. These results suggest that the interface mutants identified in this study can avoid unwanted bystander edits that are often observed with DdCBEs. To improve specificity further, we combined desired mutations to create double or triple interface mutants. Unfortunately, these mutants were either poorly efficient or inactive at the on-target site (Supplementary Fig. 1), suggesting that the resulting DddA_tox_ halves cannot interact efficiently with the other halves even when brought together on target DNA by TALE proteins. We also found that our interface-engineered DdCBE pairs, without a nuclear export signal, could nevertheless reduce or avoid off-target mutations at highly homologous sites with a single nucleotide mismatch in the nuclear pseudogenes (Supplementary Fig. 2). We performed western blotting to compare the expression levels of the interface-engineered DdCBEs to that of the wild-type DdCBE in transfected cells and found that there was no significant difference (Supplementary Fig. 3), ruling out the possibility that the engineered variants were more specific because they were poorly expressed or unstable.

### HiFi-DdCBEs avoid mitochondrial genome-wide off-target effects

**Fig. 4:**
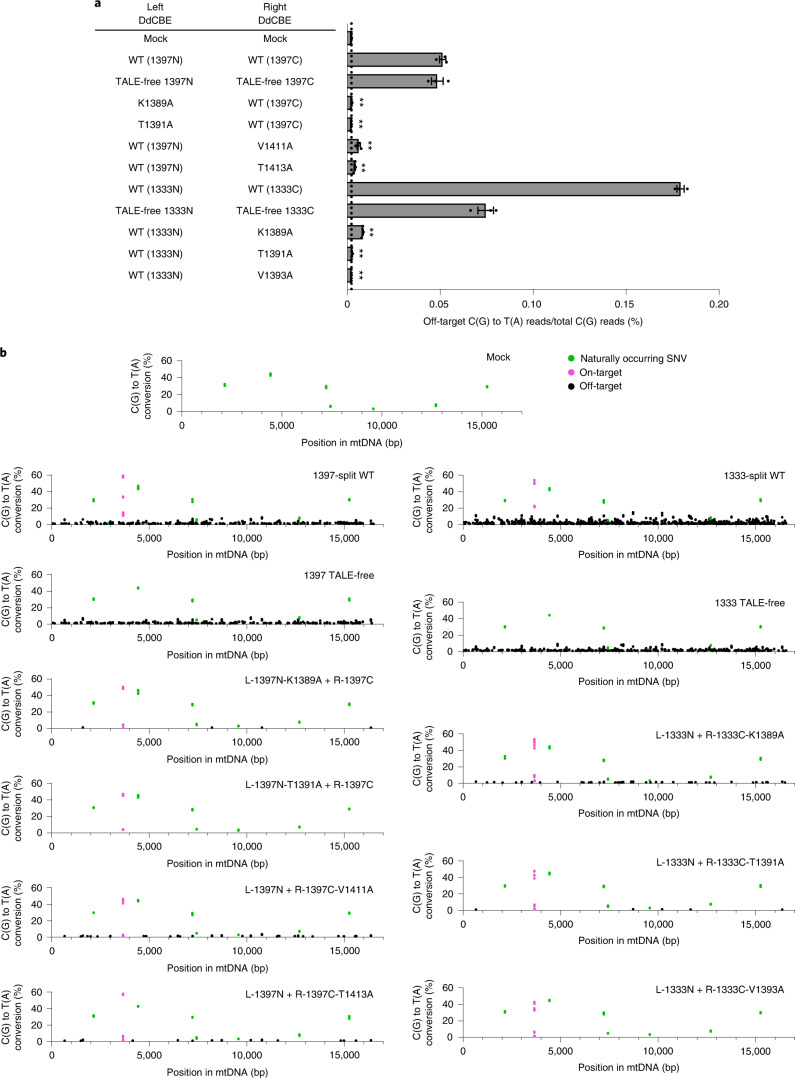
Mitochondrial genome-wide off-target editing induced by DdCBEs specific to the *MT-ND1* site. **a**, The average frequencies of mitochondrial genome-wide off-target editing induced by wild-type DdCBE, TALE-free constructs, and interface-engineered DddA_tox_ pairs. Error bars are s.e.m. for *n* = 3 biologically independent samples. *P* = 0.0010, *P* = 0.0011, *P* = 0.0002, *P* = 0.0009, *P* = 0.0001, *P* = 0.0001 and *P* = 0.0001 for L-1397N-K1389A, L-1397N-T1391A, R-1397C-V1411A, R-1397C-T1413A, R-1333C-K1389A, R-1333C-T1391A and R-1333C-V1393A compared to the wild-type pairs, respectively. **P < 0.01, Student’s two-tailed *t*-test. **b**, Mitochondrial genome-wide plots for C-to-T point mutations with frequencies ≥1%. Naturally occurring SNVs, on-target edits (including bystander edits in the editing window) and off-target edits are shown in green, magenta and black, respectively. All data points from *n* = 3 biologically independent experiments are shown. Source data

Encouraged by these results, we conducted mtDNA-wide whole-genome sequencing to investigate whether the interface mutants can reduce off-target base editing in mitochondria. We chose several interface mutants, which were highly active when paired with the wild-type partner but inefficient when paired with the TALE-free partner (K1389A and T1391A in 1397N, V1411 and T1413A in 1397C, and K1389A, T1391A and V1393A in 1333C), transfected plasmids encoding each DdCBE pair into human cells, and then analyzed mtDNA isolated from the resulting cells. High-throughput sequencing of mtDNA isolated from untreated, control cells showed the presence of several naturally occurring single-nucleotide variants (SNVs) with a heteroplasmy fraction of ≥10%. After excluding these SNVs, we calculated average frequencies of mtDNA-wide off-target C-to-T editing in each sample with frequencies ≥1%. As shown in Fig. 4a, the wild-type DdCBE pair with DddA_tox_ split at G1397 specific to the human *MT-ND1* gene induced off-target mutations with an average C-to-T editing frequency of 0.0513% ± 0.0017%, which is 22.5-fold higher than the untreated control (0.0023% ± 0.0001). Strikingly, the TALE-free, G1397-split DddA_tox_ pair also showed an average conversion frequency of 0.0485% ± 0.0031% in mtDNA, not much different from the wild-type DdCBE pair, indicating that an active deaminase can be formed by spontaneous assembly of the two inactive halves even in the absence of TALE–DNA interactions, which gives rise to collateral mutations. Furthermore, mismatched DdCBE pairs composed of a *MT-ND1* TALE and a *MT-ND4* TALE induced off-target editing with average mtDNA-wide conversion frequencies of 0.0198% ± 0.0111% (ND1-1397N + ND4-1397C) and 0.0083% ± 0.0067% (ND1-1397C + ND4-1397N), suggesting that they could form a functional DddA protein. Mismatched DdCBE pairs with a 1333-split also induced off-target editing with average conversion frequencies of 0.0513% ± 0.0199% (ND1-1333N + ND4-1333C) and 0.0261% ± 0.0161% (ND1-1333C + ND4-1333N). As expected, DdCBEs containing the catalytically deficient E1347A DddA_tox_ mutant and a single DdCBE alone without its partner, used as negative controls, did not induce off-target editing in the mitochondrial genome (Supplementary Fig. 4). Notably, the DdCBE pair containing K1389A in 1397C demonstrated an average editing frequency of 0.0023% ± 0.0002%, essentially the same as the untreated control. Likewise, the DdCBE pairs containing T1391A in 1397C, V1411A in 1397N, or T1413A in 1397N showed 8.2- to 23.3-fold reduced off-target editing, compared to the wild-type DdCBE pair, with average off-target editing frequencies of 0.0022% ± 0.0001%, 0.0062% ± 0.0009%, and 0.0041% ± 0.0003%, respectively.

We next examined the number and position of off-target edits induced by various DdCBE constructs in the mitochondrial genome (Fig. 4b). The wild-type DdCBE pair and the TALE-free, G1397-split DddA_tox_ pair caused off-target C-to-T edits at 238 and 224 sites, respectively, with conversion frequencies of ≥1.0%. Sequence logos obtained with DNA sequences around these off-target C-to-T edits showed a strong preference for the TC context (Supplementary Fig. 5), a signature of DddA_tox_ substrate specificity^1^. The majority (>80%) of these off-target sites were co-edited by the DdCBE pair and the TALE-free split DddA_tox_ pair (Supplementary Fig. 6a), indicating that DdCBE off-target editing is largely independent of TALE–DNA interactions. In sharp contrast, our interface variants induced off-target edits at merely 5 (K1389A), 0 (T1391A), 32 (V1411A) and 15 (T1413A) sites in the human mitochondrial genome. Note that the T1391A variant completely avoided off-target mutations.

We also compared the wild-type G1333-split DdCBE pair with variants containing alanine substitutions in the 1333C half. The wild-type DdCBE pair induced off-target C-to-T mutations with an average editing frequency of 0.1794% ± 0.0020%, significantly higher than that observed with the negative control. Notably, again, the TALE-free, G1333-split DddA_tox_ pair also induced off-target mutations with an average frequency of 0.0745% ± 0.0042%. As expected, interface mutants reduced such off-target, collateral mutations in mtDNA. Thus, the DdCBE pair containing K1389A, T1391A or V1393A in 1333C showed 21- to 80-fold reduced off-target editing, compared to the wild-type DdCBE pair, with average off-target editing frequencies of 0.0084% ± 0.0003%, 0.0026% ± 0.0002% and 0.0022% ± 0.00003%, respectively. Mitochondrial genome-wide plots showed that the wild-type, G1333-split DdCBE pair and the TALE-free pair induced off-target edits at 640 and 328 sites, respectively (Fig. 4b and Supplementary Fig. 6b). The vast majority (97.6%) of the 328 off-target sites edited by the TALE-free pair were also edited by the G1333-split DdCBE pair, which induced off-target edits at a total of 320 additional sites. Presumably, these additional off-target edits were caused by nonspecific TALE–DNA interactions. DdCBE pairs containing our interface variants induced off-target edits at merely 51 (K1389A), 5 (T1391A) and 0 (V1393A) sites, largely or completely avoiding these collateral mutations.

### Precision base editing in human mitochondrial genes with HiFi-DdCBEs

**Fig. 5:**
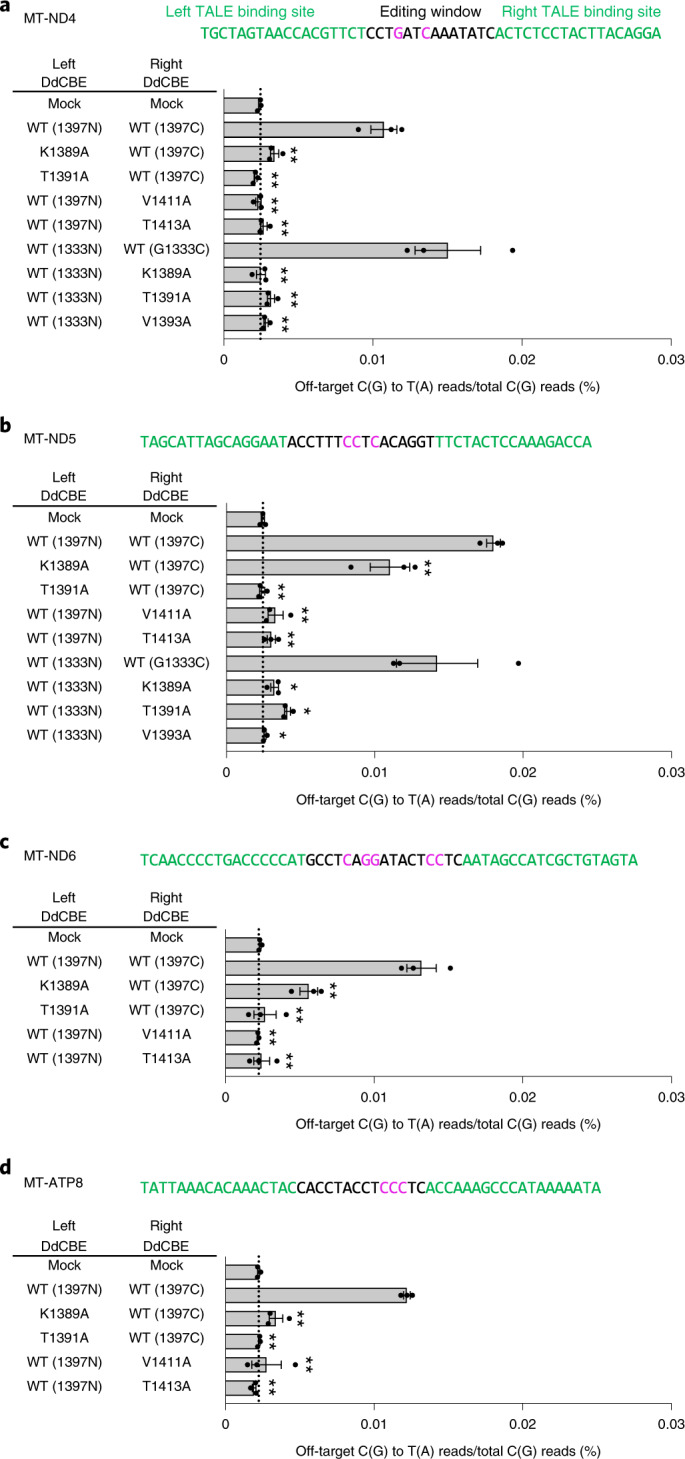
Mitochondrial genome-wide off-target analysis for DdCBEs specific to four different human mtDNA sites. **a**–**d**,The *MT-ND4* (**a**), *MT-ND5* (**b**), *MT-ND6* (**c**) and *MT-ATP8* (**d**) sites targeted by DdCBE and interface-engineered variants. The bar graphs indicate the average frequencies of mitochondrial genome-wide off-target editing by wild-type and engineered DddA_tox_ pairs. Error bars are s.e.m. for *n* = 3 biologically independent samples. *P < 0.05 and **P < 0.01, Student’s two-tailed *t*-test. Exact *P* values are provided in the source data. Source data

We next chose four different regions in the human mitochondrial genome (*MT-ND4*, *MT-ND5*, *MT-ND6*and *MT-ATP8*) to investigate whether our interface-engineered, HiFi-DdCBEs can achieve efficient C-to-T conversions at the four target sites without inducing collateral mutations (Fig. 5). As expected, the wild-type DdCBEs specific to these sites induced off-target mutations with average C-to-T editing frequencies that ranged from 0.0107% ± 0.0009% (*MT-ND4*) to 0.0180% ± 0.0005% (*MT-ND5*), which were 4.5- to 7.3-fold higher than than that for the untreated control (0.0024% ± 0.0001%). HiFi-DdCBEs containing interface-engineered DddA_tox_ halves, however, largely avoided off-target base editing (Fig. 5 and Supplementary Figs. 7–10). For example, the *ND4*-specific DdCBE pair with the T1391A mutation in 1397N exhibited an average off-target editing efficiency of 0.0021% ± 0.0001%, a 5.0-fold (0.0107%/0.0021%) reduction, compared to the wild-type DdCBE. The pair with the K1389A mutation in 1333C showed a 6.0-fold (0.0150%/0.0025%) reduction, compared to the wild-type pair. For the other target sites (Fig. 5b–d), our HiFi-DdCBEs also demonstrated reduced off-target editing; for example, with average frequencies of 0.0110% (K1389A at MT*-ND5*) and 0.0024% (T1391A at MT*-ND5*) for the G1397-split system and 0.0041% (T1391A at MT*-ND5*) and 0.0026% (V1393A at MT*-ND5*) for the G1333-split system. In particular, HiFi-DdCBEs containing the T1391A variant specific to *MT-ND4* or the T1413A variant specific to *MT-ATP8* did not induce any off-target C-to-T edits in the human mitochondrial genome with frequencies of ≥1.0%, whereas wild-type DdCBEs induced a total of 63 and 86 off-target edits, respectively. The on-target editing efficiencies of these HiFi-DdCBEs were comparable with those of the wild-type DdCBEs (Supplementary Fig. 11).

**Fig. 6:**
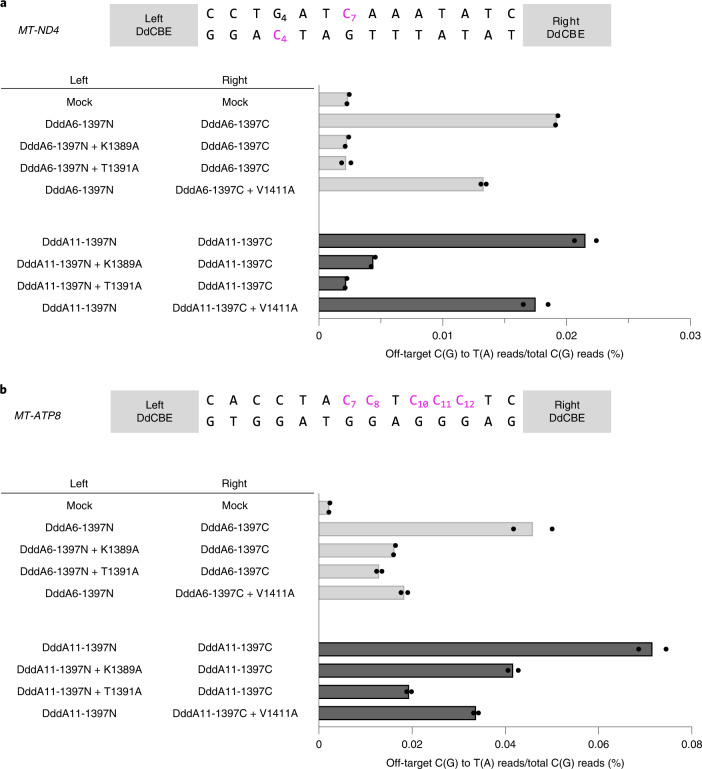
Mitochondrial genome-wide off-target analysis for DddA6, and DddA11 and their HiFi variants. **a**,**b**, The average frequencies of mitochondrial genome-wide off-target editing induced by DddA6/DddA11 and interface-engineered variants targeting *MT-ND4* (**a**) *and MT-ATP8* (**b**) sites for *n* = 2 biologically independent samples. The editing windows are shown at the top and target cytosine bases are in magenta. Source data

Last, we introduced the interface mutations in DddA6 and DddA11, evolved DddA variants^21^, to create HiFi-DdCBEs with enhanced activity and broadened targeting scope, respectively. We found that DdCBEs containing DddA6 induced off-target mutations in the mitochondrial genome with average C-to-T editing frequencies that ranged from 0.0192% ± 0.0001% (*MT-ND4*) to 0.0459% ± 0.0041% (*MT-ATP8*), which were 8.1- to 20-fold higher than that for the untreated control (0.0024% ± 0.0001%). By contrast, HiFi-DdCBEs containing DddA6 largely avoided off-target base editing (Fig. 6 and Supplementary Figs. 12, 13). Thus, the *ND4*-specific DddA6-containing CBE pair with the K1389A mutation exhibited an average off-target editing efficiency of 0.0023% ± 0.0001%, an 8.5-fold (0.0192%/0.0023%) reduction, compared to the wild-type DddA6-containing pair. The DdCBE pair with the T1391A mutation in DddA6 also showed an 8.7-fold (0.0192%/0.0022%) reduction, compared to the DddA6-containing CBE pair. The *ATP8*-specific DddA6-containing CBE pair with the K1389A mutation exhibited an average off-target editing frequency of 0.0162% ± 0.0001%, a 2.8-fold (0.0459%/0.0162%) reduction compared to the wild-type DddA6-containing CBE pair. Likewise, the DdCBE pair with T1391A or V1411A in DddA6 demonstrated a 3.5-fold (0.0459%/0.0130%) or a 2.5-fold (0.0459%/0.0184%) reduction respectively, compared to the wild-type DddA6-containing CBE pair.

Furthermore, HiFi-DdCBEs containing DddA11 with interface mutations also largely avoided off-target base editing. DddA11-containing DdCBEs specific to two sites induced off-target mutations with average editing frequencies that ranged from 0.0215% ± 0.0009% (*MT-ND4*) to 0.0716% ± 0.0029% (*MT-ATP8*), which were 9.1- to 32-fold higher than that of the untreated control. By contrast, *MT-ND4*-specific HiFi-DdCBEs with interface mutations were highly specific, with average off-target editing frequencies of 0.0044% ± 0.0002% (K1389A), 0.0022% ± 0.0001% (T1391A) and 0.0175% ± 0.0010% (V1411A). Thus, these HiFi-DdCBEs showed a 4.9-fold (0.0215%/0.0044%), 9.8-fold (0.0215%/0.0022%) and 1.2-fold (0.0215%/0.0175%) reduction, respectively, compared to the DddA11-containing DdCBEs. Likewise, *MT-ATP8*-specific HiFi-DdCBEs with interface mutations demonstrated average off-target editing frequencies of 0.0417% ± 0.0011% (K1389A), 0.0194% ± 0.0005% (T1391A) and 0.0337% ± 0.0010% (V1411A). Thus, these HiFi-DdCBEs showed a 1.7-fold (0.0716%/0.0417%), 3.7-fold (0.0716%/0.0194%) and 2.1-fold (0.0716%/0.0337%) reduction, respectively, compared to the DddA11-containing DdCBEs. On-target editing efficiencies of these HiFi-DdCBEs were comparable with those of the wild-type DdCBE pairs containing DddA6 or DddA11 (Supplementary Fig. 14). Taken together, these results demonstrate that DdCBE off-target base editing in the mitochondrial genome can be largely avoided using our HiFi-DdCBEs with interface-engineered DddA_tox_ split constructs.

## Discussion

We found that conventional DdCBEs induce up to hundreds of off-target mutations in human mtDNA, which are probably caused by spontaneous assembly of split DddA_tox_ halves even in the absence of TALE–DNA interactions. To avoid collateral mutations, we developed HiFi-DdCBEs, in which amino acid residues in the split dimer interface were mutated to disrupt the formation of the functional deaminase in the absence of TALE–DNA interactions. Very recently, Lei et al. reported that DdCBEs could induce off-target mutations at both TALE-dependent and TALE-independent sites in the nuclear genome and that these nuclear off-target mutations could be largely avoided by fusing a nuclear export signal to DdCBEs^14^. Our preliminary data suggest that HiFi-DdCBEs, without a nuclear export signal, could nevertheless avoid TALE-independent off-target mutations (Supplementary Fig. 15) as well as TALE-dependent off-target mutations in the nuclear genome (Supplementary Fig. 2). We recommend the use of the T1391A variant in 1397N or 1333C when high specificity is desired over high activity, and the use of the K1389A variant in 1397N or 1333C when high activity is desired over high specificity. Note that *MT-ND1*-specific, 1397-split or 1333-split HiFi-DdCBEs with K1389A were as active as wild-type DdCBEs targeted to the same sites but were much more specific, showing a 22-fold or 21-fold difference, respectively, in average mtDNA-wide off-target editing frequencies (Fig. 4). This result suggests that the high specificity of HiFi-DdCBEs does not necessarily stem from reduced activity. These interface variations can also improve the specificity of DddA6 or DddA11 to create HiFi-DdCBEs with enhanced activity or expanded targeting scope, respectively. In this study, HiFi-DdCBEs were transiently expressed in transfected cells. It will be important to investigate whether HiFi-DdCBEs would still be able to avoid generating off-target effects in the long term, when stably transfected. In conclusion, HiFi-DdCBEs will have broad utility for making disease models in cell lines and animals, and for correcting pathogenic mtDNA mutations associated with mitochondrial genetic disorders in patients.

## Methods

### Plasmid construction

We introduced point mutations into DdCBE expression plasmids through site-directed mutagenesis. In brief, we amplified plasmids with mutagenesis primers before mutagenesis with the Q5 Site-Directed Mutagenesis Kit (NEB), and the results were confirmed with Sanger sequencing. For assembly of plasmids encoding interface mutant DdCBEs, the mini-prepped mutant expression plasmid was added to a solution containing module vectors (each encoding a TALE array^22^), BsaI-HFv2 (10 U), T4 DNA ligase (200 U) and buffer in a single tube. For the TALE-free construct, instead of module vectors we used oligonucleotides (Macrogen) (forward, 5′-CTGAGTGGTAGTGGTAGTGGTTCTGG-3′; reverse, 5′-ACCGCCAGAACCACTACCACTACCAC-3′) that encode a flexible linker N-(SG)_3_-C. amino acid sequences of the TALE arrays for each target are indicated in Supplementary Fig. 16. The restriction and ligation reactions were performed in a thermocycler, with 20 cycles at 37 °C and 16 °C for 5 min each, followed by final incubations at 50 °C for 15 min and 80 °C for 5 min. Ligated plasmids were chemically transformed into *Escherichia coli* DH5ɑ; plasmids from the transformants were subjected to Sanger sequencing to analyze the identity of the constructs. Final plasmids were midi-prepped (Qiagen) for cell transfection.

### Mammalian cell culture and transfection

HEK293T/17 cells (CRL-11268, American Type Culture Collection) were cultured and maintained at 37 °C in 5% CO_2_. Cells were grown in DMEM supplemented with 10% (v/v) fetal bovine serum (Gibco) in the absence of antibiotics. Cells were not tested for mycoplasma contamination. For lipofection, cells were plated in 48-well cell culture plates (SPL) at a density of 5 × 10^4^ cells per well, 18–24 h before transfection. Lipofection using Lipofectamine 2000 (Invitrogen) was performed with 500 ng of each TALE half monomer plasmid to make up 1,000 ng of total plasmid DNA. Cells were collected on day four post transfection.

### Western blot

Cell extracts were prepared by scraping the cells with 60–80 μl of lysis buffer (20 mM HEPES, pH 7.4, 150 mM NaCl, 10% glycerol, 1% Triton X-100, 1 mM PMSF, 10 μg ml^−1^ of leupeptin, 10 μg ml^−1^ of aprotinin, 1 mM Na3VO4, 1 mM NaF and 10 mM β-glycerophosphate) at 4 °C and stored on ice for 10 min. the supernatants were collected by centrifugation at 12,000×*g* for 10 min at 4 °C. The concentration of cleared lysate was measured by the Bradford assay (Bio-Rad), protein lysate was loaded onto 10% acrylamide gel, and the gel transferred to a PVDF membrane (Bio-Rad). The membrane was incubated in blocking solution containing 5% nonfat dried milk in TBS-T for 1 h at room temperature (25 °C) and subsequently incubated with primary antibody (diluted at a ratio of 1:2,000) in TBS-T (20 mM Tris, 150 mM NaCl, and 0.1 % Tween 20) at 4 °C overnight. Then the membrane was washed four times in TBS-T and incubated in blocking solution containing secondary antibody conjugated with HRP (diluted at a ratio of 1:5,000) for 1 h at room temperature. The membrane was further washed four times in TBS-T, and specific protein complexes were visualized with the ECL Plus substrate (GE Healthcare). The antibodies used were: anti-FLAG tag (SAB4301135, Sigma Aldrich), anti-HA tag (ab215069, Abcam), b-actin (sc-47778), anti-mouse (sc-2005), anti-rabbit (sc-2004) and anti-goat (sc-2020) (Santa Cruz).

### Genomic and mitochondrial DNA isolation for high-throughput sequencing

In preparation for isolation of genomic DNA from cultured cells, the culture medium was aspirated and a lysis buffer containing proteinase K from the DNeasy Blood & Tissue Kit (Qiagen) was added to detach cells from the culture plates. Genomic DNA was extracted following the manufacturer’s protocol. 1 µl of lysate was used as a template for high-throughput sequencing. For whole mitochondrial genome sequencing, purified genomic DNA was amplified with two sets of primers (hWGS1-F, hWGS1-R, hWGS2-F and hWGS2-R in Supplementary Table 2), using Takara LA Taq (Takara) for long-fragment PCR. Both PCR products were purified using AMPure XP (Beckman Coulter) following the manufacturer’s protocol.

### High-throughput sequencing

To create a targeted deep-sequencing library, nested PCR was performed, and final index sequences were incorporated using Q5 DNA Polymerase. The library was subjected to paired-end read sequencing using MiniSeq (Illumina). For whole mitochondrial genome sequencing, amplified mtDNA was subjected to tagmentation using a DNA prep kit (Illumina) following the manufacturer’s protocol. The paired-end sequencing results were joined into a single fastqjoin file for deep-sequencing and analyzed using CRISPR RGEN Tools (http://www.rgenome.net)^23^. Targeted deep sequencing was carried out at a sequencing depth of >8,000⨉. Whole mitochondrial genome sequencing data were assembled by Minimap2 using Geneious Prime. The variants with ≥1% conversion rates and ≥30 Q scores were used. The average read depth for mtDNA-wide analysis was 3,100⨉. For data visualization, we used Microsoft Excel, Microsoft PowerPoint and Prism 9. Source data are provided with this paper.

### Reporting summary

Further information on research design is available in the Nature Research Reporting Summary linked to this article.

## Online content

Any methods, additional references, Nature Research reporting summaries, source data, extended data, supplementary information, acknowledgements, peer review information; details of author contributions and competing interests; and statements of data and code availability are available at 10.1038/s41587-022-01486-w.

## Supplementary information

Supplementary Information Supplementary Figs. 1–16 and Tables 1–3.

Reporting Summary

Supplementary Data 1 Statistical Source data for supplementary figures.

Supplementary Data 2 Uncropped blot images for Supplementary figure S3.

## Data Availability

All data supporting the results are available in the main text or supplementary materials. All data that support the findings of this study are available from the corresponding author upon request. The high-throughput sequencing data from this study have been deposited in the NCBI Sequence Read Archive (SRA) database under the accession codes PRJNA817018 and PRJNA847381. Source data are provided with this paper.

## Source data

Source Data Fig. 2 Statistical Source Data
Source Data Fig. 3 Statistical Source Data
Source Data Fig. 4 Statistical Source Data
Source Data Fig. 5 Statistical Source Data
Source Data Fig. 6 Statistical Source Data

## Code availability

Python code used in this study is described in the supplementary information. Source data are provided with this paper.
