# Supplementary material for: Precision mitochondrial DNA editing with high-fidelity DddA-derived base editors
Source: Nat Biotechnol. 2022 Oct 13;41(3):378–86. doi: 10.1038/s41587-022-01486-w (PMC10017512; doi:10.1038/s41587-022-01486-w)
Supplement: Supplementary file 1 — Supplementary Figs. 1–16 and Tables 1–3. [file 41587_2022_1486_MOESM1_ESM.pdf]

nature biotechnology

Article https://doi.org/10.1038/s41587-022-01486-w

Precision mitochondrial DNA editing with high-fidelity DddA-derived base editors

In the format provided by the authors and unedited

Supporting Information

Precision mitochondrial DNA editing with high-fidelity DddA-derived base editors

Seonghyun Lee[1], Hyunji Lee[2], Gayoung Baek[1], and Jin-Soo Kim[1]

[1] Center for Genome Engineering, Institute for Basic Science, Daejeon 34126, Republic of Korea

[2] Laboratory Animal Resource Center, Korea Research Institute of Bioscience and Biotechnology, Yeongudanji-ro 30, Cheongju 28116, Korea

Correspondence should be addressed to J.-S.K. (jskim01@snu.ac.kr).

Contents

Supplementary Table S1. Primers used for mutagenesis
Supplementary Table S2. Primers for high-throughput sequencing
Supplementary Table S3. Python code for PyMOL
Supplementary Figure S1. Effects of multiple mutations in HiFi-DdCBEs
Supplementary Figure S2. Off-target editing efficiencies of DdCBEs in nuclear pseudogenes
Supplementary Figure S3. Expression levels of DdCBE and interface-engineered mutants examined using western blot analysis
Supplementary Figure S4. Mitochondrial genome-wide off-target editing induced by DdCBEs in control and experimental setups
Supplementary Figure S5. Sequence logos that reveal the sequence context of off-target edits induced by wild-type DdCBE and the interface-engineered HiFi-DdCBEs
Supplementary Figure S6. Venn diagrams showing the overlap between off-target sites edited by DdCBEs and TALE-free constructs
Supplementary Figure S7. Mitochondrial genome-wide plots showing mutations induced by wild-type and HiFi-DdCBEs specific for the *MT-ND4* target
Supplementary Figure S8. Mitochondrial genome-wide plots showing mutations induced by wild-type and HiFi-DdCBEs specific for the *MT-ND5* target
Supplementary Figure S9. Mitochondrial genome-wide plots showing mutations induced by wild-type and HiFi-DdCBEs specific for the *MT-ND6* target
Supplementary Figure S10. Mitochondrial genome-wide plots showing mutations induced by wild-type and HiFi-DdCBEs specific for the *MT-ATP8* target
Supplementary Figure S11. Heatmaps showing editing efficiencies induced by DdCBE and the various mutants at the *MT-ND4*, *MT-ND5*, *MT-ND6*, and *MT-ATP8* target sites
Supplementary Figure S12. Mitochondrial genome-wide plots showing mutations induced by wild-type DddA6/11 and HiFi-DdCBEs specific for the MT-ND4 target
Supplementary Figure S13. Mitochondrial genome-wide plots showing mutations induced by wild-type DddA6/11 and HiFi-DdCBEs specific for the MT-ATP8 target
Supplementary Figure S14. On-target efficiency using DddA6 and DddA11
Supplementary Figure S15. TALE-independent off-target editing
Supplementary Figure S16. Sequences of TALE arrays

Supplementary Table 1. Primers used for mutagenesis

Name	Sequence
Left-1333N_F1315A_F	AGTGGGGACCGCATACTATGTCAACGAC
Left-1333N_F1315A_R	GTCTGACCGTTGTAGGCG
Left-1333N_F1329A_F	ATCCAAGGTTGCATCTAGCGGTGGGTCCGG
Left-1333N_F1329A_R	TCCAATCCCCCGGCGTCG
Left-1333N_G1313A_F	CAGACAGTGGCAACCTTTTACTATGTCAACGAC
Left-1333N_G1313A_R	ACCGTTGTAGGCGGGGAG
Left-1333N_I1299A_F	TCCATATCAGGCAAGTGCTCCCCAACTCCC
Left-1333N_I1299A_R	CCCAGGGCGTAGGAACCA
Left-1333N_K1327A_F	ATTGGAATCCGCAGTTTTCTCTAGCGGTGGGTCCGG
Left-1333N_K1327A_R	CCCCCGGCGTCGTTGACA
Left-1333N_L1294A_F	CTCTGGTTCCTACGCCGCAGGTCCATATCAGATTAG
Left-1333N_L1294A_R	CTAATCTGATATGGACCTGCGGCGTAGGAACCAGAG
Left-1333N_S1300A_F	ATATCAGATTGCAGCTCCCCAACTCCCCGC
Left-1333N_S1300A_R	GGACCCAGGGCGTAGGAA
Left-1333N_S1330A_F	CAAGGTTTTCGCAAGCGGTGGGTC
Left-1333N_S1330A_R	GATTCCAATCCCCCGGCG
Left-1333N_T1314A_F	GGTCAGACAGTGGGGGCATTTTACTATGTC
Left-1333N_T1314A_R	GACATAGTAAAATGCCCCCACTGTCTGACC
Left-1333N_V1312A_F	GGTCAGACAGCAGGGACCTTTTACTATG
Left-1333N_V1312A_R	GTTGTAGGCGGGGAGTTG
Left-1333N_V1318A_F	TTTTACTATGCAAACGACGCCGGGG
Left-1333N_V1318A_R	GGTCCCCACTGTCTGACC
Left-1333N_Y1292A_F	CTCTGGTTCCGCAGCCCTGGGTCCATATCAGATTAGTGCTCC
Left-1333N_Y1292A_R	CCAGAGCCCCCCAACCCC
Left-1333N_Y1316A_F	CAGTGGGGACCTTTGCATATGTCAACGACGC
Left-1333N_Y1316A_R	GCGTCGTTGACATATGCAAAGGTCCCCACTG
Left-1333N_Y1317A_F	GACCTTTTACGCAGTCAACGACGCCGGG
Left-1333N_Y1317A_R	CCCACTGTCTGACCGTTG
Left-1397N_C1376A_F	TGTGGCTTCGCAGTGAATATGACTGAAACCCTTCTG
Left-1397N_C1376A_R	CATATTCACTGCGAAGCCACAAGTACCCTCTG
Left-1397N_E1381A_F	GTGTGAATATGACTGCAACCCTTCTGCCCGAAAATG
Left-1397N_E1381A_R	CATTTTCGGGCAGAAGGGTTGCAGTCATATTCACAC
Left-1397N_K1389A_F	GAAAATGCCGCAATGACTGTCGTCCCACCTGAAGG
Left-1397N_K1389A_R	GACAGTCATTGCGGCATTTTCGGGCAGAAGG
Left-1397N_L1384A_F	GAAACCCTTGCACCCGAAAATGCCAAGATGAC
Left-1397N_L1384A_R	ATTTTCGGGTGCAAGGGTTTCAGTCATATTCACACAGAAG
Left-1397N_M1390A_F	AATGCCAAGGCAACTGTCGTCCCACCTGAAGG
Left-1397N_M1390A_R	GACGACAGTTGCCTTGGCATTTTCGGGCAGAAGG
Left-1397N_T1380A_F	GTGAATATGGCAGAAACCCTTCTGCCCGAAAATG
Left-1397N_T1380A_R	AAGGGTTTCTGCCATATTCACACAGAAGCCACAAG

Left-1397N_T1391A_F	CGAAAATGCCAAGATGGCAGTCGTCCCACCTGAAG
Left-1397N_T1391A_R	CTTCAGGTGGGACGACTGCCATCTTGGCATTTTCG
Left-1397N_V1377A_F	GGTACTTGTGGCTTCTGTGCAAATATGACTGAAACC
Left-1397N_V1377A_R	GGTTTCAGTCATATTTGCACAGAAGCCACAAGTACC
Left-1397N_V1392A_F	GAAAATGCCAAGATGACTGCAGTCCCACCTGAAGG
Left-1397N_V1392A_R	CCTTCAGGTGGGACTGCAGTCATCTTGGCATTTTC
Right-1333C_E1361A_F	GGCATTAGCGCAGGGCTTGTGTTCCATAATAATCC
Right-1333C_E1361A_R	CACAAGCCCTGCGCTAATGCCGTTATCTCTCATAAAAAGAGC
Right-1333C_F1353A_F	GTCAGCTCTTGCAATGAGAGATAACGGCATTAGCGAAGGG
Right-1333C_F1353A_R	TGCCCCTCGACGTGCCCG
Right-1333C_F1365A_F	GGGCTTGTGGCACATAATAATCCTGAGGGCACCTG
Right-1333C_F1365A_R	GATTATTATGTGCCACAAGCCCTTCGCTAATG
Right-1333C_G1362A_F	CATTAGCGAAGCACTTGTGTTCC
Right-1333C_G1362A_R	CCGTTATCTCTCATAAAAAG
Right-1333C_H1366A_F	GCTTGTGTTCGCAAATAATCCTGAGGGCAC
Right-1333C_H1366A_R	CCTTCGCTAATGCCGTTA
Right-1333C_K1389A_F	TGAGAACGCTGCAATGACTGTCG
Right-1333C_K1389A_R	GGCAGAAGTGTTTCGGTC
Right-1333C_L1363A_F	TAGCGAAGGGGCAGTGTTCCATAATAATCCTGAGGGC
Right-1333C_L1363A_R	ATGGAACACTGCCCCTTCGCTAATGCCGTTATCTC
Right-1333C_M1354A_F	AGCTCTTTTTGCAAGAGATAACGGCATTAGCGAAGG
Right-1333C_M1354A_R	GACTGCCCCTCGACGTGC
Right-1333C_M1390A_F	GAACGCTAAAGCAACTGTCGTACCAC
Right-1333C_M1390A_R	TCAGGCAGAAGTGTTTCG
Right-1333C_Q1349A_F	CGTCGAGGGGGCATCAGCTCTTTTTATGAGAGATAACGGC
Right-1333C_Q1349A_R	TGCCCGGCGTTAGCGTAG
Right-1333C_S1350A_F	CGAGGGGCAGGCAGCTCTTTTTATG
Right-1333C_S1350A_R	ACGTGCCCGGCGTTAGCG
Right-1333C_T1391A_F	CGCTAAAATGGCAGTCGTACCAC
Right-1333C_T1391A_R	TTCTCAGGCAGAAGTGTTTC
Right-1333C_V1346A_F	GCCGGGCACGCAGAGGGGCAGT
Right-1333C_V1346A_R	GTTAGCGTAGTTAGGATAAGGTGTTG
Right-1333C_V1364A_F	CGAAGGGCTTGCATTCCATAATAATCC
Right-1333C_V1364A_R	CTAATGCCGTTATCTCTC
Right-1333C_V1393A_F	AATGACTGTCGCACCACCCGAAG
Right-1333C_V1393A_R	TTAGCGTTCTCAGGCAGAAG
Right-1397C_F1412A_F	ACAAAGGTGGCAACAGGCAACTCTAACAGTCCAAAG
Right-1397C_F1412A_R	GTTGCCTGTTGCCACCTTTGTCTCCCCTGTTGCTCC
Right-1397C_K1410A_F	CAACAGGGGAGACAGCAGTGTTCACAGGCAA
Right-1397C_K1410A_R	TTGCCTGTGAACACTGCTGTCTCCCCTGTTG
Right-1397C_T1413A_F	GGAGACAAAGGTGTTCGCAGGCAACTCTAACAGTCC
Right-1397C_T1413A_R	GGACTGTTAGAGTTGCCTGCGAACACCTTTGTCTCC
Right-1397C_V1411A_F	GAGACAAAGGCATTCACAGGCAACTCTAACAGTC

Right-1397C_V1411A_R	GCCTGTGAATGCCTTTGTCTCCCCTGTTGCTCC
DddA6-K1389A-F	GGAAAACGCCGCAATGACTGTCGTCCC
DddA6-K1389A-R	GGCAGCAGGGTCTCGATC
DddA11-K1389A-F	CGAAAATGCCGCAATGACTGTCGTC
DddA11-K1389A-R	GGCAGAAGGGTTTCAATC
DddA6-T1391A-F	CGCCAAGATGGCAGTCGTCCCAC
DddA6-T1391A-R	TTTTCCGGCAGCAGGGTC
DddA11-T1391A-F	TGCCAAGATGGCAGTCGTCCCAC
DddA11-T1391A-R	TTTTCGGGCAGAAGGGTTTC
DddA6/11-V1411A-F	GAGACAAAGGCCTTCATAGGCAACTCTAACAGTCCAAAGAG
DddA6/11-V1411A-R	CCCTGTTGCTCCCCGCTT

Supplementary Table S2. Primers for high-throughput sequencing

Name	Sequence
hND1_1st_F	CTAAAACCCGCCACATCTACC
hND1_1st_R	TCGGCTATGAAGAATAGGGCG
hND1_2nd_F	ACACTCTTTCCCTACACGACGCTCTTCCGATCTCCTCAACCTAGGCCTCCTATTT
hND1_2nd_R	GTGACTGGAGTTCAGACGTGTGCTCTTCCGATCTTGAGATTGTTTGGGCTACTGC
hND4_1st_F	CATTGCATACTCTTCAATCAGCC
hND4_1st_R	GGATAGGTGTATGAACATGAGG
hND4_2nd_F	ACACTCTTTCCCTACACGACGCTCTTCCGATCTCAAGGACTTCAAACTCTACTCCC
hND4_2nd_R	GTGACTGGAGTTCAGACGTGTGCTCTTCCGATCTAATATGTAGAGGGAGTATAGGGC
hND5_1st_F	CTCTAACACTATGCTTAGGCG
hND5_1st_R	CGTTAATGTTAGTAAGGGTGGG
hND5_2nd_F	ACACTCTTTCCCTACACGACGCTCTTCCGATCTCCATACCTCTCACTTCAACC
hND5_2nd_R	GTGACTGGAGTTCAGACGTGTGCTCTTCCGATCTGCGATGAGAGTAATAGATAGGGC
hND6_1st_F	CTTTCTTCTTCCCACTCATCC
hND6_1st_R	GAGTGTGGGTTTAGTAATGGG
hND6_2nd_F	ACACTCTTTCCCTACACGACGCTCTTCCGATCTTAACCCCACTAAAACACTCAC
hND6_2nd_R	GTGACTGGAGTTCAGACGTGTGCTCTTCCGATCTATTTTGGGGGAGGTTATATGGG
hATP8_1st_F	CATCGTCCTAGAATTAATTCCCC
hATP8_1st_R	AGTATAAGAGATCAGGTTCGTCC
hATP8_2nd_F	ACACTCTTTCCCTACACGACGCTCTTCCGATCTTCCTTACACTATTCCTCATCACC
hATP8_2nd_R	GTGACTGGAGTTCAGACGTGTGCTCTTCCGATCTGGGAAATAGAATGATCAGTACTGC
hWGS1-F	AAAGCACATACCAAGGCCAC
hWGS1-R	TTGGCTCTCCTTGCAAAGTT
hWGS2-F	TATCCGCCATCCCATACATT
hWGS2-R	AATGTTGAGCCGTAGATGCC
nuND4_1st_F	TAGTCGCCCACGGACTTAC
nuND4_1st_R	GGATAGGAGGAGGATAGGGGATA
nuND4_2nd_F	ACACTCTTTCCCTACACGACGCTCTTCCGATCTCAAGCCTCACTAATCTCGCC
nuND4_2nd_R	GTGACTGGAGTTCAGACGTGTGCTCTTCCGATCTTTAATGTGGTGGCTGAGCG
nuND5_1st_F	CTCTCACACTATGCCTAGGCG
nuND5_1st_R	CGTTAATGTTAGTGAGGGCTGG
nuND5_2nd_F	ACACTCTTTCCCTACACGACGCTCTTCCGATCTGGACTACTCAAAACCATACCTCTT
nuND5_2nd_R	GTGACTGGAGTTCAGACGTGTGCTCTTCCGATCTGGTGGCGATGAGAGTAATAGAC
nuND6_1st_F	ACACTCTTTCCCTACACGACGCTCTTCCGATCTCACTAATCCTACTTCCATCACCAA
nuND6_1st_R	GTGACTGGAGTTCAGACGTGTGCTCTTCCGATCTTCTGAATTACGGGGGAGGTTAC
nuND6_2nd_F	GTCCTTACCAGATTAGCGCACCCCAACTCCCAGCATACAATGGTCGGAC
nuND6_2nd_R	GCCTTCAGGTGGGACGACAGTCATCTTGGCGTTTTCCGGCAGCAGGG
ND6-OT1_1st_F	TCCCTGCACTTGTCAATCATT
ND6-OT1_1st_R	AAGGCTCTCTTCAGTGTCAGT
ND6-OT1_2nd_F	ACACTCTTTCCCTACACGACGCTCTTCCGATCTGGAGAAACGTCACGGTATGC
ND6-OT1_2nd_R	GTGACTGGAGTTCAGACGTGTGCTCTTCCGATCTGGTGGTCAACAATTCTCACTGC
ND6-OT2_1st_F	CCCTCCCTTATGTATGTTGCA
ND6-OT2_1st_R	GGGGTGAAAACTGGAGCTAAA
ND6-OT2_2nd_F	ACACTCTTTCCCTACACGACGCTCTTCCGATCTTGCGAGGCTCAAGTAAGTAGT
ND6-OT2_2nd_R	GTGACTGGAGTTCAGACGTGTGCTCTTCCGATCTACGAAGGGTTCCGCATTG
ND6-OT3_1st_F	AGGAAACCTCTTGCTGGAATT
ND6-OT3_1st_R	CCTTTCCCCTACACTGCTATC
ND6-OT3_2nd_F	ACACTCTTTCCCTACACGACGCTCTTCCGATCTACTTATAGTCGGAGCAAGCA
ND6-OT3_2nd_R	GTGACTGGAGTTCAGACGTGTGCTCTTCCGATCTACGATTGCTCAGTCTCTCC
ND6-OT4_1st_F	CATCACCAAATGTCCCAGGTC

ND6-OT4_1st_R	GCCACCAGAGCCTAGAAATAG
ND6-OT4_2nd_F	ACACTCTTTCCCTACACGACGCTCTTCCGATCTGTACTAAGAGGATGACGAGGC
ND6-OT4_2nd_R	GTGACTGGAGTTCAGACGTGTGCTCTTCCGATCTAGATCATTTGTAAGGGAAGCCG
ND6-OT5_1st_F	AAGTCCCTCTCTCAACAATTGG
ND6-OT5_1st_R	GCACAGGCTTCAGAGTCATAA
ND6-OT5_2nd_F	ACACTCTTTCCCTACACGACGCTCTTCCGATCTTGTCCGCAGAACTACAGAGG
ND6-OT5_2nd_R	GTGACTGGAGTTCAGACGTGTGCTCTTCCGATCTCGCAAAGCCATAGTTCAGGG

Supplementary Table S3. Python code for PyMOL

We adopted Python code for PyMOL from PyMOL wiki (https://pymolwiki.org/index.php/InterfaceResidues).

```
>
from pymol import stored

def interfaceResidues(cmpx, cA='c. A', cB='c. B', cutoff=1.0, selName="interface"):
    oldDS = cmd.get("dot_solvent")
    cmd.set("dot_solvent", 1)
    tempC, selName1 = "tempComplex", selName+"1"
    chA, chB = "chA", "chB"
    cmd.create(tempC, cmpx)
    cmd.disable(cmpx)
    cmd.remove(tempC + " and not (polymer and (%s or %s))" % (cA, cB))
    cmd.get_area(tempC, load_b=1)
    cmd.alter(tempC, 'q=b')
    cmd.extract(chA, tempC + " and (" + cA + ")")
    cmd.extract(chB, tempC + " and (" + cB + ")")
    cmd.get_area(chA, load_b=1)
    cmd.get_area(chB, load_b=1)
    cmd.alter( "%s or %s" % (chA,chB), "b=b-q" )
    stored.r, rVal, seen = [], [], []
    cmd.iterate('%s or %s' % (chA, chB), 'stored.r.append((model,resi,b))')

    cmd.enable(cmpx)
    cmd.select(selName1, None)
    for (model,resi,diff) in stored.r:
        key=resi+"-"+model
        if abs(diff)>=float(cutoff):
            if key in seen: continue
            else: seen.append(key)
            rVal.append( (model,resi,diff) )
            cmd.select( selName1, selName1 + " or (%s and i. %s)" % (model,resi))

    cmd.select(selName, cmpx + " in " + selName1)
    cmd.delete(selName1)
    cmd.delete(chA)
    cmd.delete(chB)
    cmd.delete(tempC)

    cmd.enable(selName)
    cmd.set("dot_solvent", oldDS)

    return rVal

cmd.extend("interfaceResidues", interfaceResidues)
```

Supplementary Figure S1. Effects of multiple mutations in HiFi-DdCBEs. Combinations of screened mutations in the (a) 1397 split and (b) 1333 split systems are shown. The annotated amino acid changes were introduced into Left-DdCBE or Right-DdCBE. Bar graphs show the mean of n = 2 biologically independent replicates.

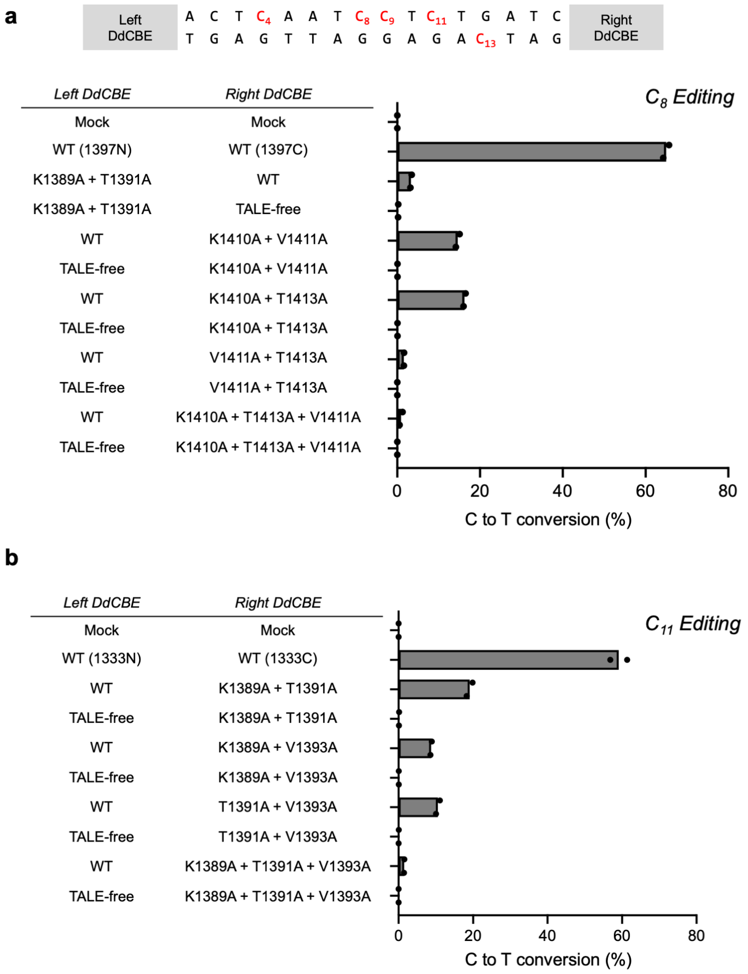

Supplementary Figure S2. Off-target editing efficiencies of DdCBEs in nuclear pseudogenes. On-target editing sites in mitochondrial DNA (mtDNA) and the corresponding nuclear pseudogene (nuDNA) with similar TALE binding sites are shown for (a) *MT-ND4*, (b) *MT-ND5*, and (c) *MT-ND6*. TALE binding sequences are shown in green, nucleotides that are mismatched between the mtDNA and nuDNA are in red, and target bases for C-to-T conversion are in magenta. Bar graphs show the mean of n = 2 biologically independent samples.

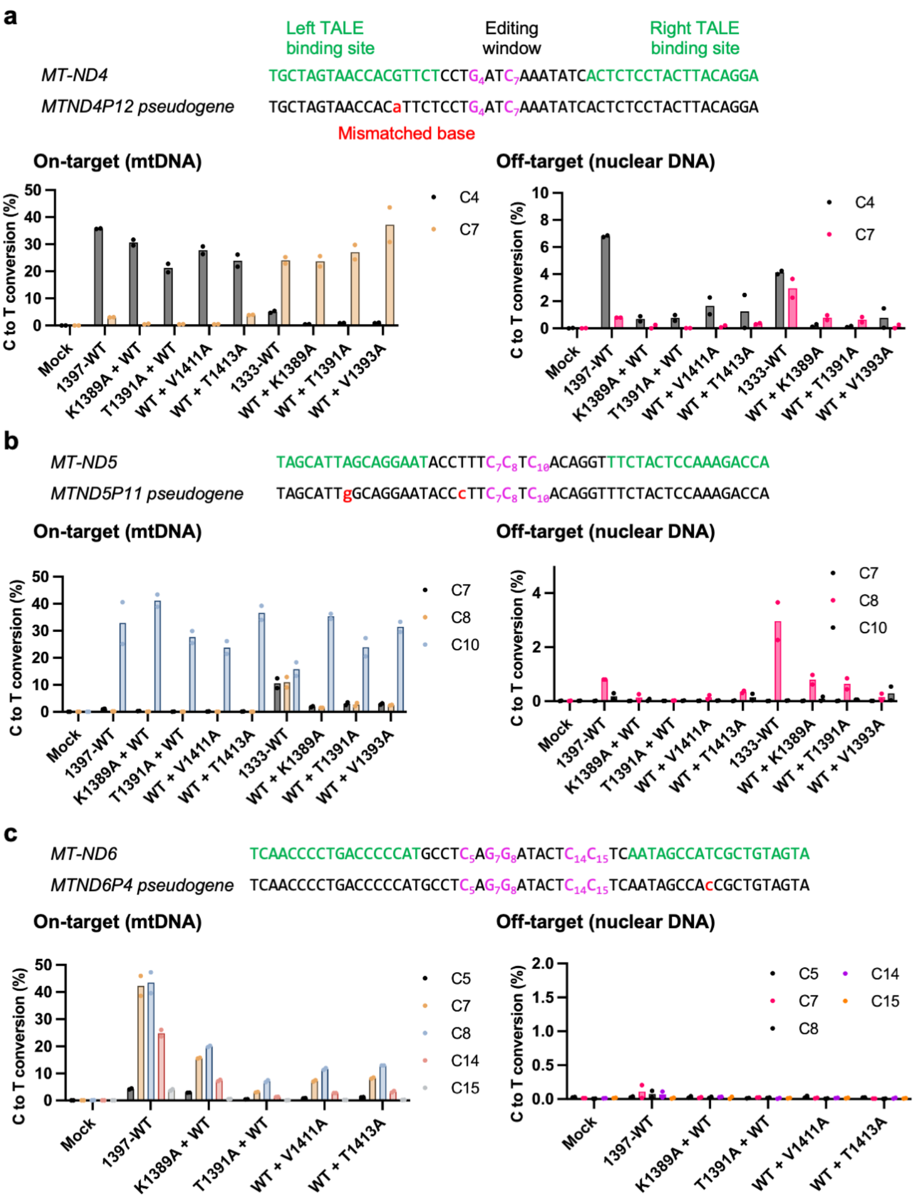

Supplementary Figure S3. Expression levels of DdCBE and interface-engineered mutants examined using western blot analysis. The transfected DdCBE pairs are indicated at the top of the blot. Left DdCBE was detected using anti-HA antibodies, and Right DdCBE was detected using anti-FLAG antibodies.

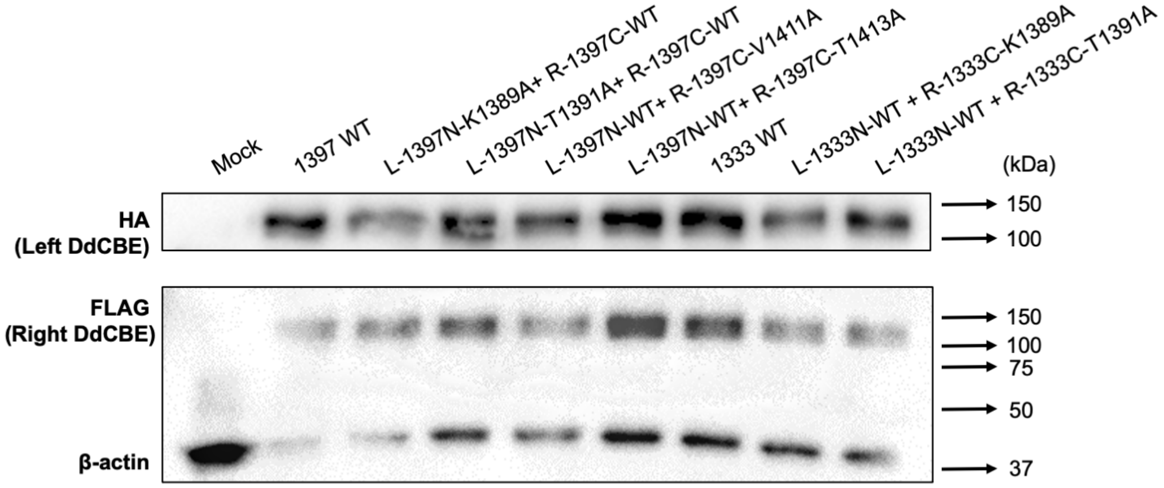

Supplementary Figure S4. Mitochondrial genome-wide off-target editing induced by DdCBEs in control and experimental setups. Frequencies of off-target edits induced by wild-type DdCBE pairs, DdCBE pairs containing the dead E1347A $DddA_{tox}$ mutant, mismatched DdCBE pairs containing *MT-ND1* and *MT-ND4* TALE binding sequences, and unpaired DdCBEs are shown for the 1397 split system (Top) and the 1333 split system (Bottom). Bar graphs show the mean of n = 3 biologically independent replicates. Error bars represent s.e.m.

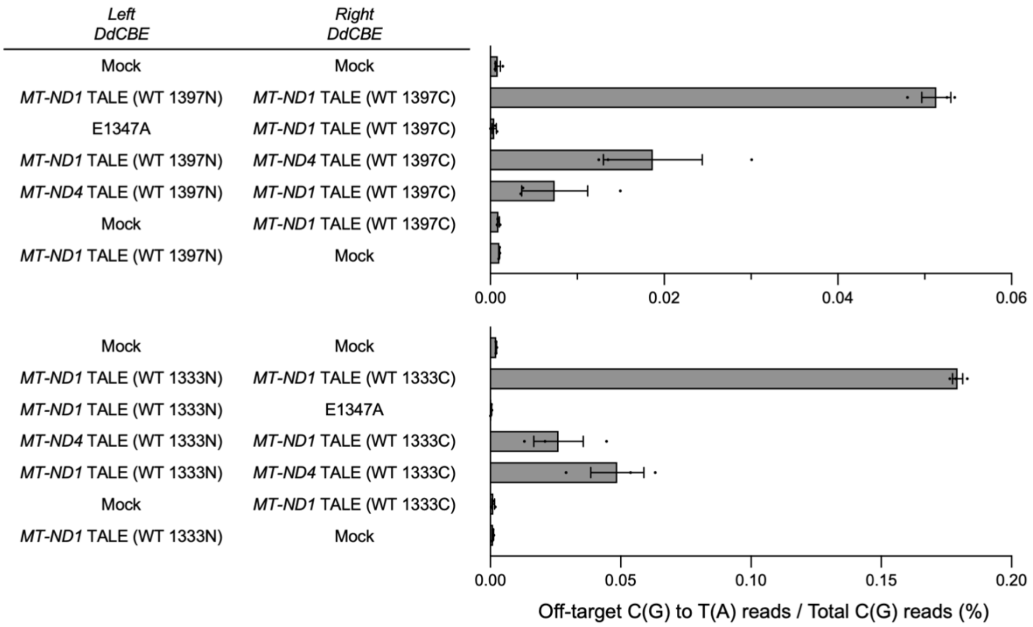

Supplementary Figure S5. Sequence logos that reveal the sequence context of off-target edits induced by wild-type DdCBE and the interface-engineered HiFi-DdCBEs

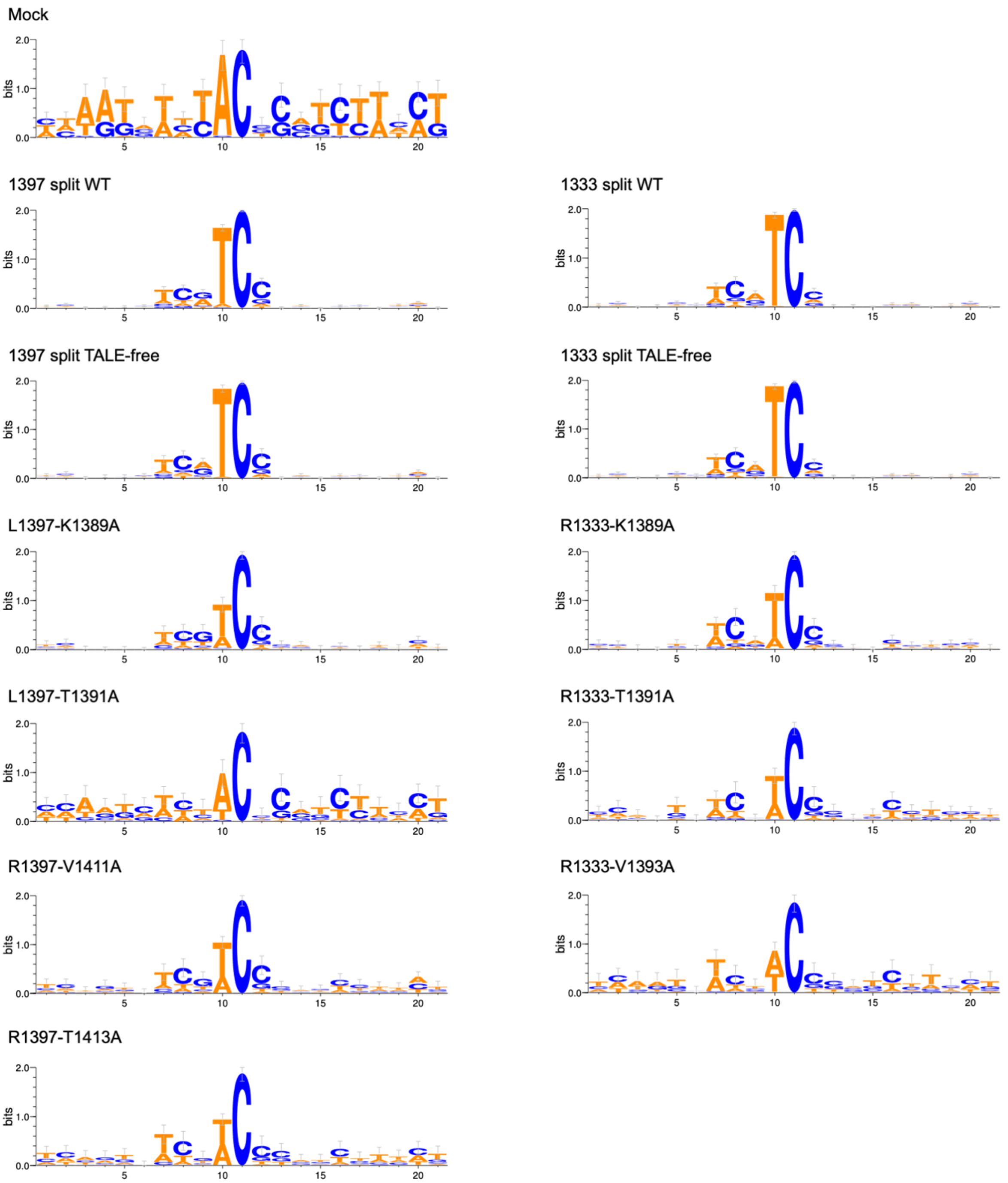

Supplementary Figure S6. Venn diagrams showing the overlap between off-target sites edited by DdCBEs and TALE-free constructs. Off-target sites edited by (a) the wild-type DdCBE pair with $DddA_{tox}$ split at G1397 and (b) G1333 targeting the *MT-ND1* site compared with sites edited by the corresponding TALE-free split $DddA_{tox}$ pairs. The numbers of annotated mutations are a summary of the data from the n = 3 biologically independent samples used in Fig. 4.

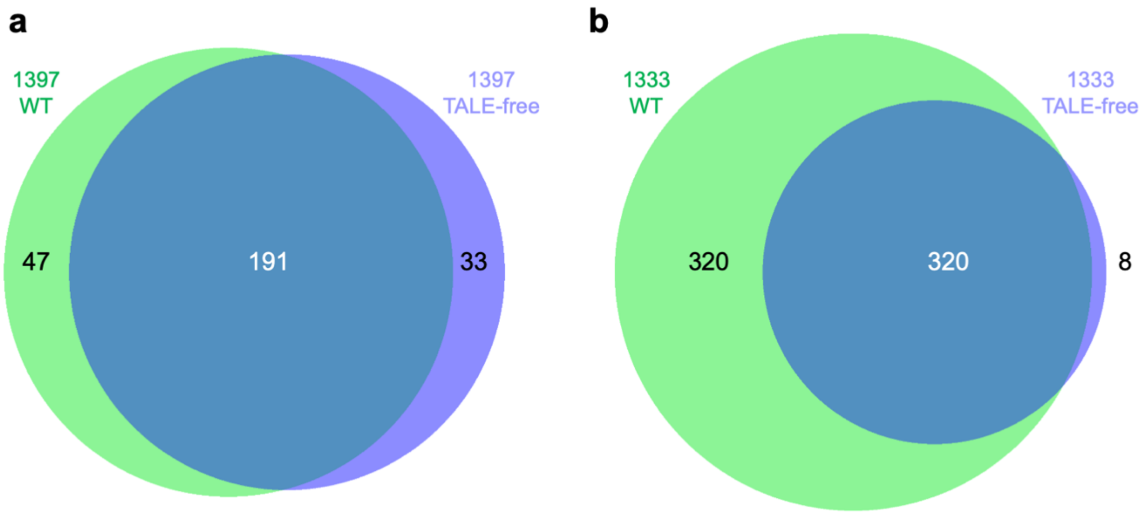

Supplementary Figure S7. Mitochondrial genome-wide plots showing mutations induced by wild-type and HiFi-DdCBEs specific for the *MT-ND4* target.

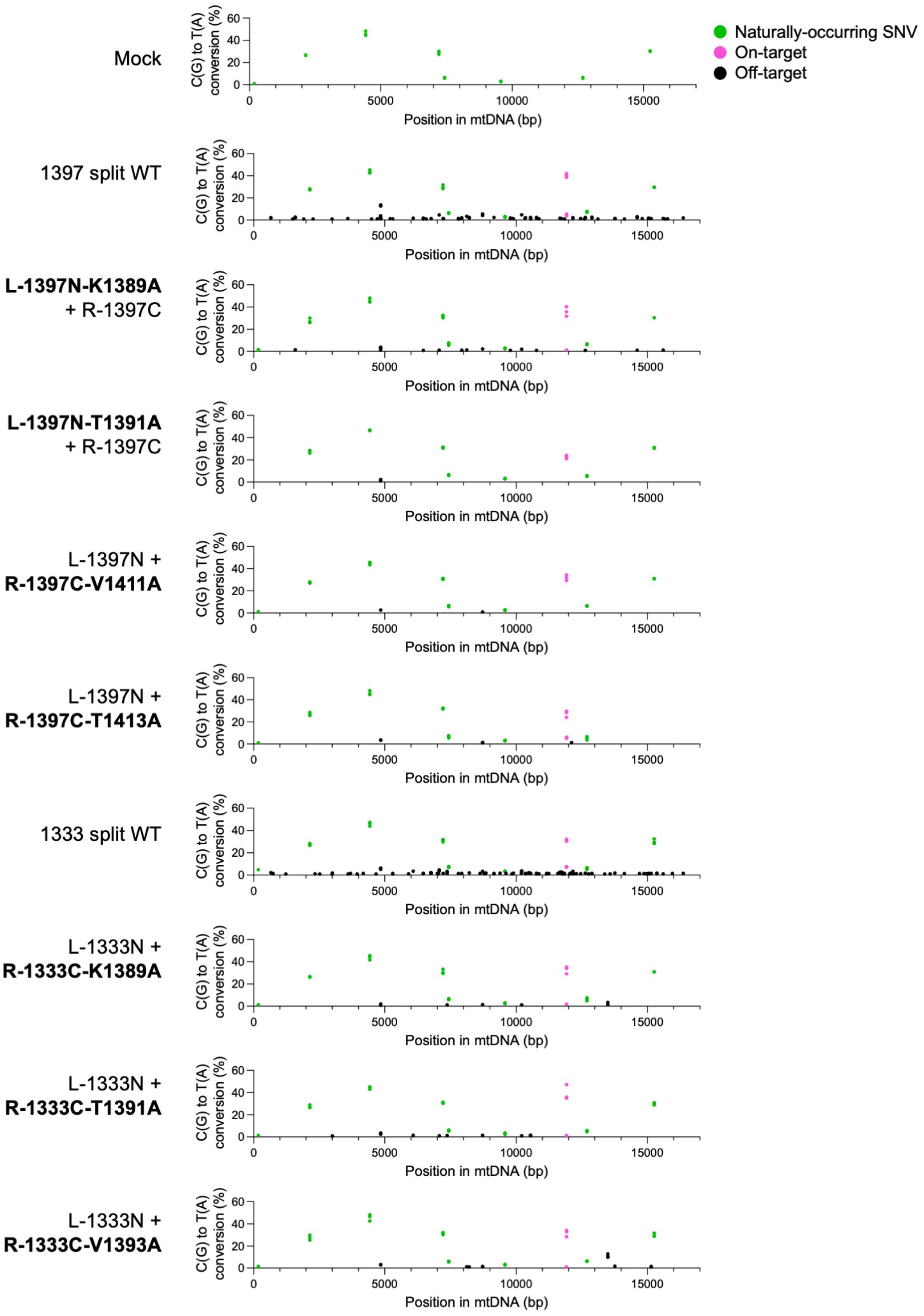

Supplementary Figure S8. Mitochondrial genome-wide plots showing mutations induced by wild-type and HiFi-DdCBEs specific for the *MT-ND5* target.

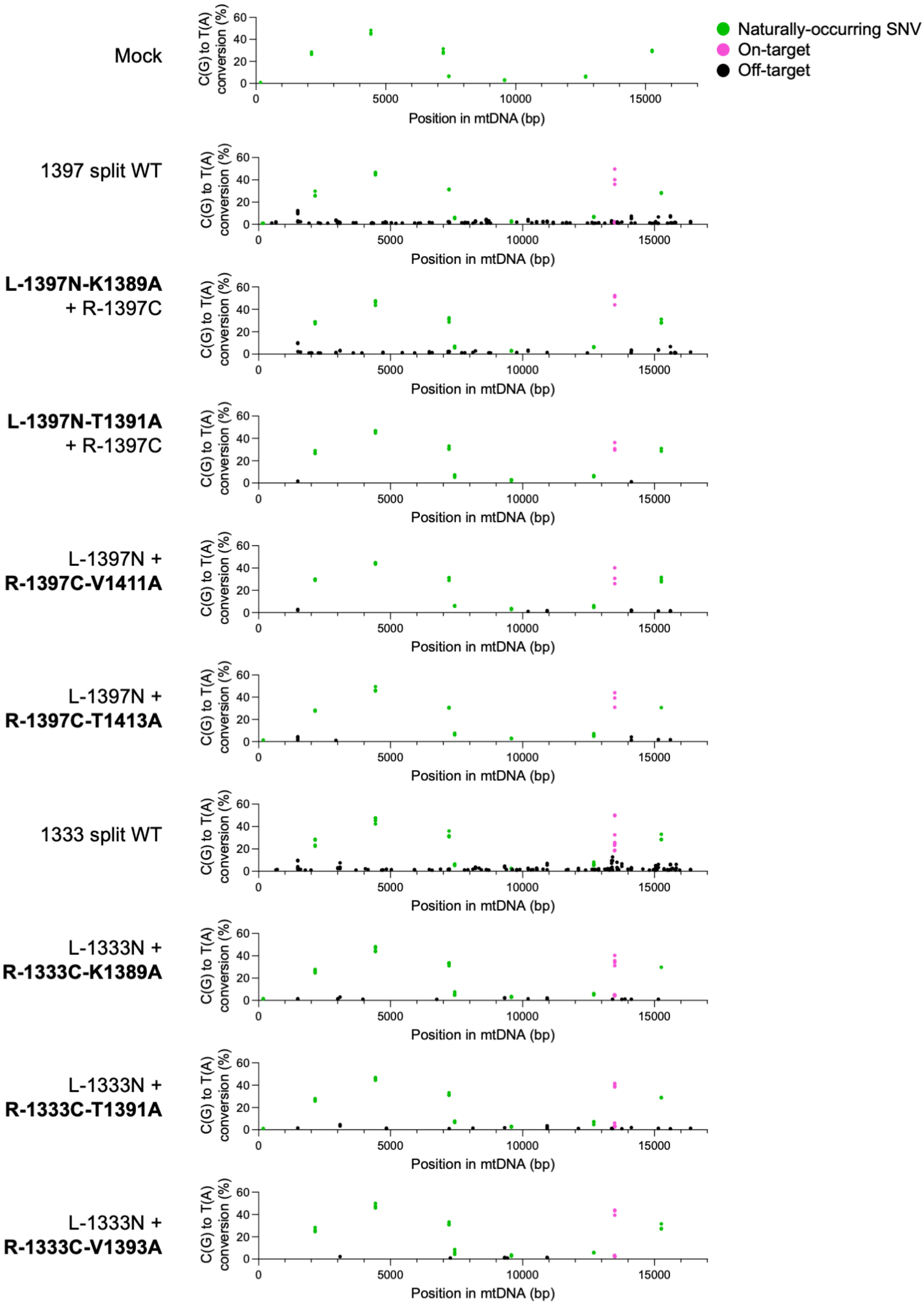

Supplementary Figure S9. Mitochondrial genome-wide plots showing mutations induced by wild-type and HiFi-DdCBEs specific for the *MT-ND6* target.

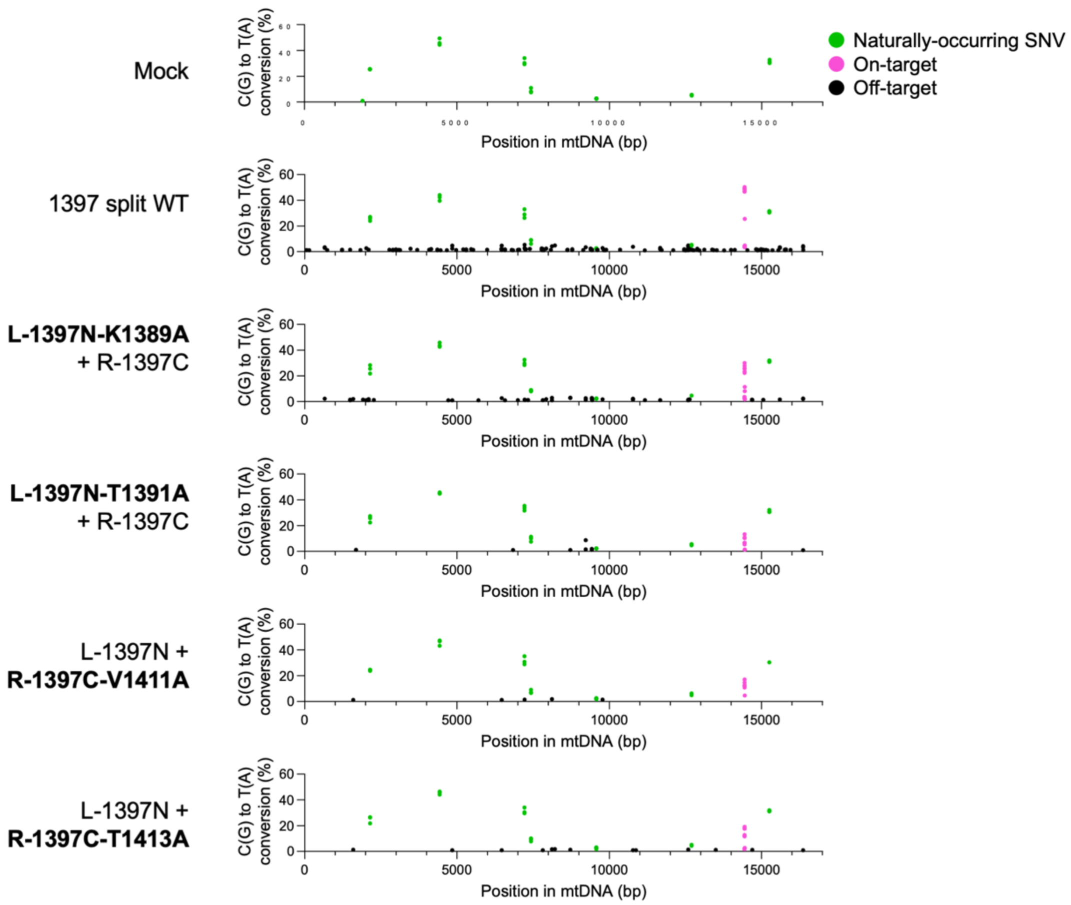

Supplementary Figure S10. Mitochondrial genome-wide plots showing mutations induced by wild-type and HiFi-DdCBEs specific for the *MT-ATP8* target.

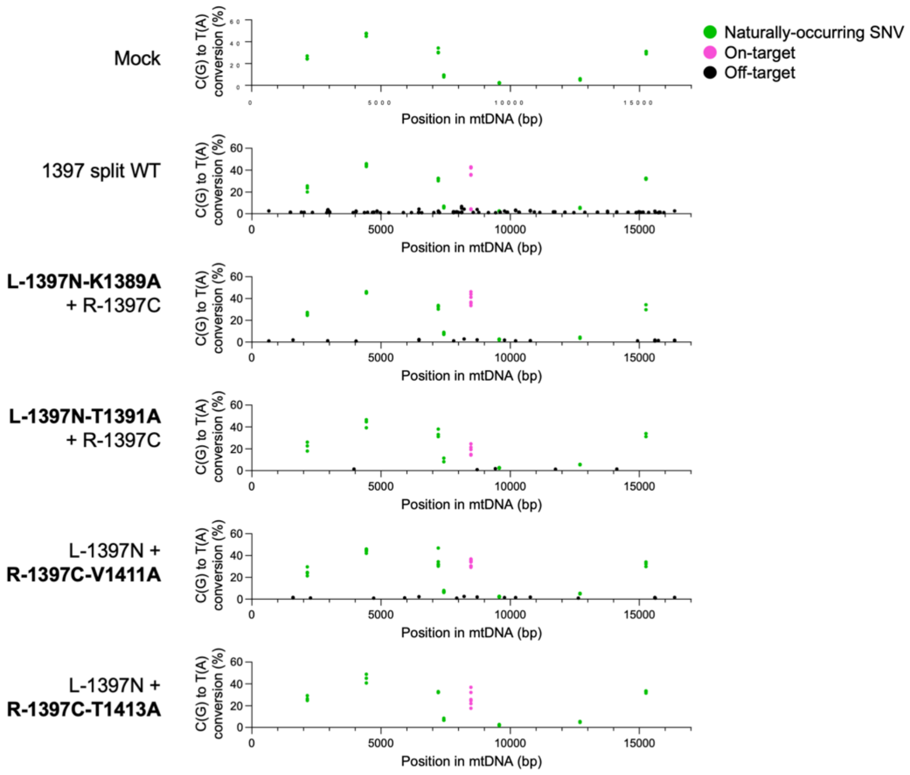

Supplementary Figure S11. Heatmaps showing editing efficiencies induced by DdCBE and the various mutants at the (a) *MT-ND4*, (b) *MT-ND5*, (c) *MT-ND6*, and (d) *MT-ATP8* target sites.

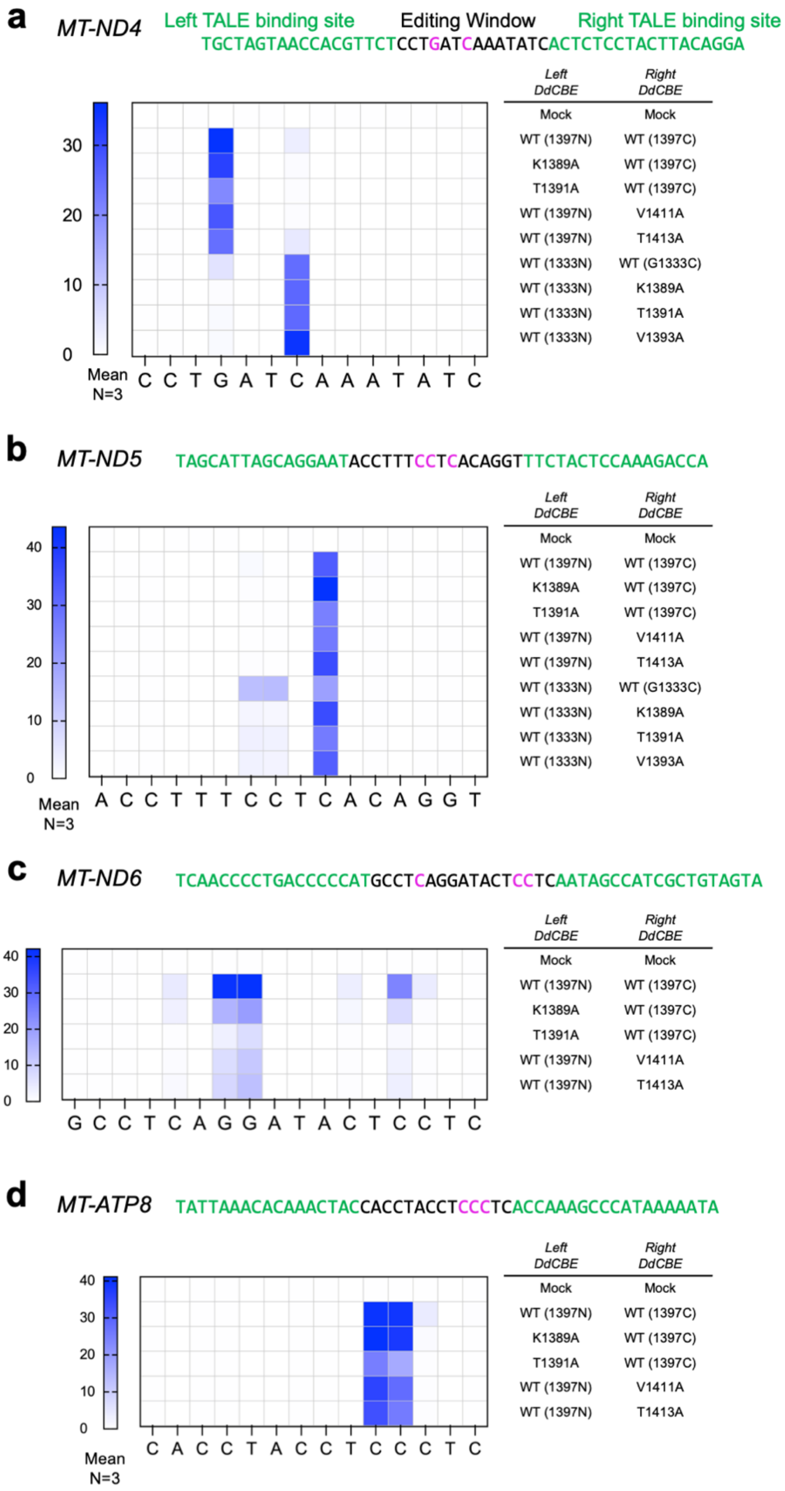

Supplementary Figure S12. Mitochondrial genome-wide plots showing mutations induced by wild-type DddA6/11 and HiFi-DdCBEs specific for the *MT-ND4* target.

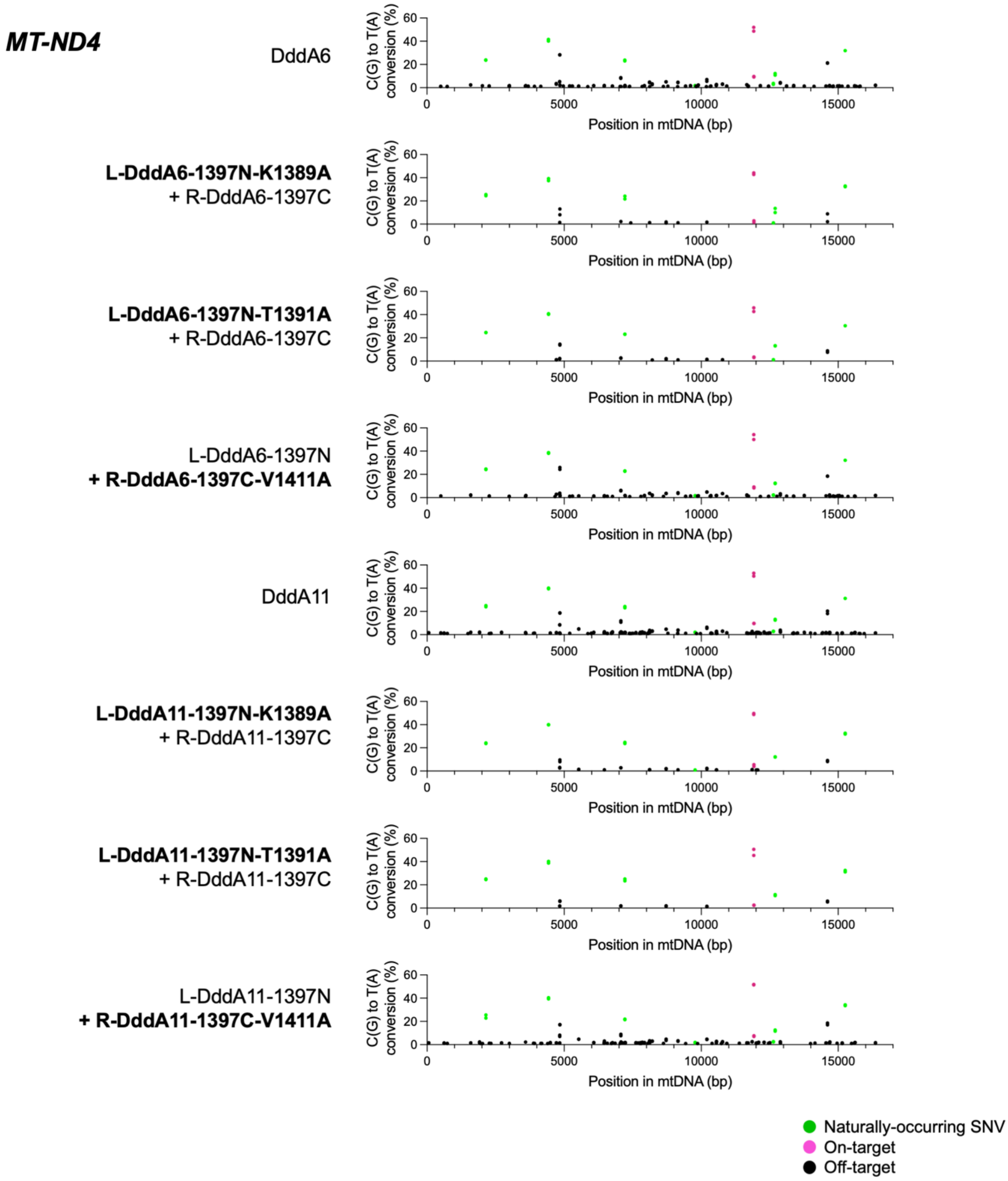

Supplementary Figure S13. Mitochondrial genome-wide plots showing mutations induced by wild-type DddA6/11 and HiFi-DdCBEs specific for the *MT-ATP8* target.

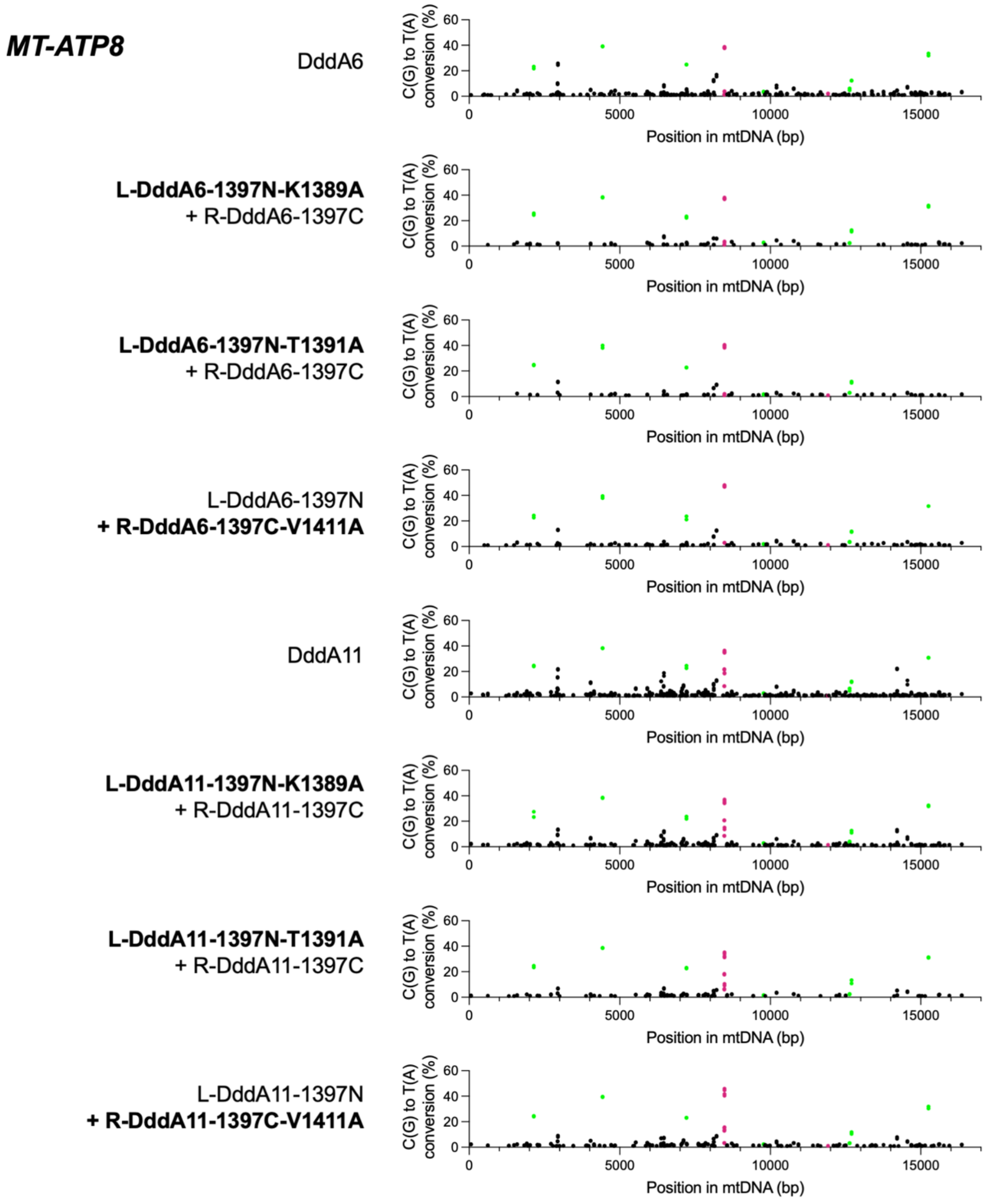

Supplementary Figure S14. On-target editing efficiencies induced using DddA6 and DddA11. Average editing efficiencies are shown at the (a) *MT-ND4* and (b) *MT-ATP8* sites. Bar graphs show the mean of n = 2 biologically independent samples. The editing windows are shown at the top of the graphs and target cytosine bases are shown in magenta.

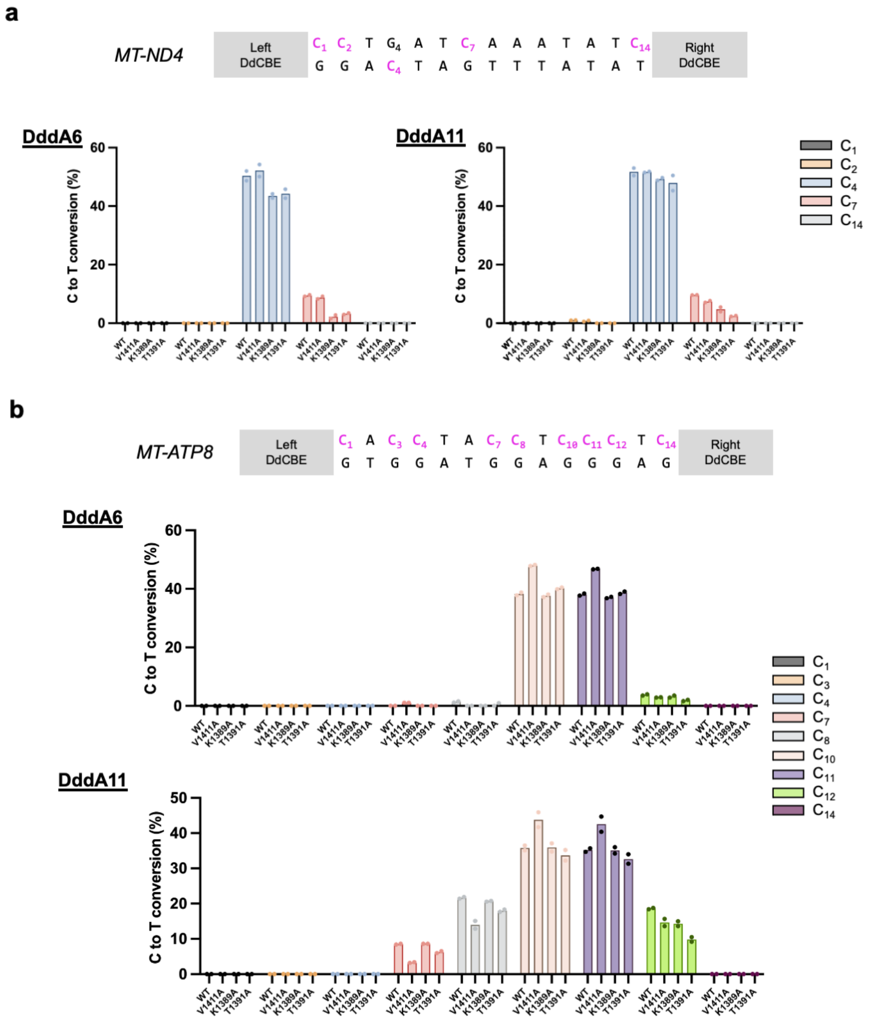

.

Supplementary Figure S15. Editing efficiencies of the 1937-split DdCBE or HiFi-DdCBEs targeted to MT-ND6 at TALE-independent off-target sites in the nuclear genome. TALE-independent off-target sites were reported by Lei et al. (2022)[14]. Bar graphs show the mean of n = 3 biologically independent samples with error bars for s.e.m.

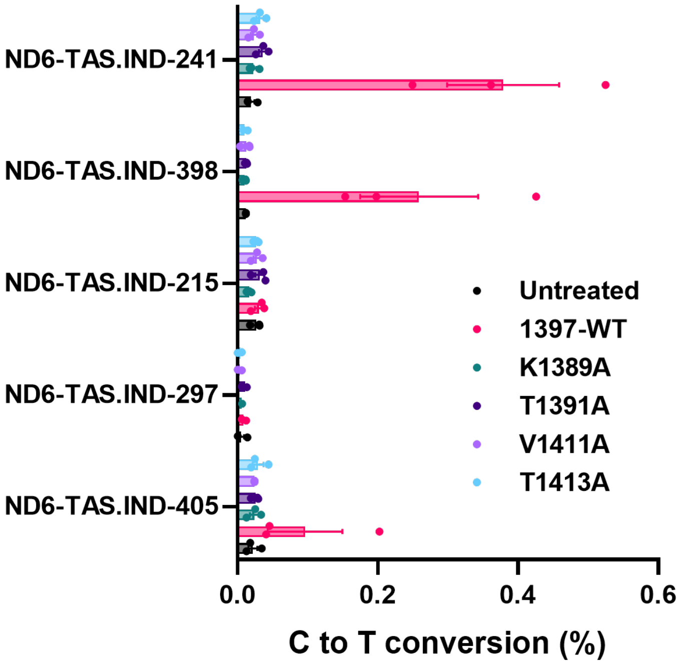

Supplementary Figure S16. Sequences of TALE arrays

N-terminal domain of TALE

GIRIQDLRTLGYSQQQQEKIKPKVRSTVAQHHEALVGHGFTHAHIVALSQHPAALGTVAVKYQDMIAA
LPEATHEAIVGVGKQWSGARALEALLTVAGELRGPPLQLDTGQLLKIAKRGGVTAVEAVHAWRNALT
GAPL

C-terminal domain of TALE

LTPEQVVAIASNGGGKQALESIVAQLSRPDPALAALTNDHLVALACLGGRPALDAVKKGLGGS

ND1 Left TALE

NLTPDQVVAIAS**HD**GGKQALETVQRLLPVLCQAHGLTPDQVVAIAS**NG**GGKQALETVQRLLPVLCQD
HGLTPAQVVAIAS**NI**GGKQALETVQRLLPVLCQDHGLTPDQVVAIAS**NN**GGKQALETVQRLLPVLCQA
HGLTPDQVVAIAS**HD**GGKQALETVQRLLPVLCQDHGLTPDQVVAIAS**HD**GGKQALETVQRLLPVLCQ
AHGLTPAQVVAIAS**NG**GGKQALETVQRLLPVLCQDHGLTPAQVVAIAS**NI**GGKQALETVQRLLPVLCQ
AHGLTPDQVVAIASN**NG**GKQALETVQRLLPVLCQAHGLTPAQVVAIAS**HD**GGKQALETVQRLLPVLC
QDHGLTPDQVVAIAS**HD**GGKQALETVQRLLPVLCQAHGLTPEQVVAIAS**NN**GGKQALETVQRLLPVL
CQAHGLTPAQVVAIAS**NG**GGKQALETVQRLLPVLCQAHGLTPAQVVAIAS**NG**GGKQALETVQRLLPV
LCQAHG

ND1 Right TALE

NLTPDQVVAIAS**NN**GGKQALETVQRLLPVLCQDHGLTPAQVVAIAS**NI**GGKQALETVQRLLPVLCQAH
GLTPEQVVAIAS**NN**GGKQALETVQRLLPVLCQAHGLTPDQVVAIAS**NG**GGKQALETVQRLLPVLCQA
HGLTPAQVVAIAS**NG**GGKQALETVQRLLPVLCQAHGLTPEQVVAIAS**NG**GGKQALETVQRLLPVLCQ
AHGLTPAQVVAIAS**NN**GGKQALETVQRLLPVLCQDHGLTPAQVVAIAS**NI**GGKQALETVQRLLPVLCQ
AHGLTPEQVVAIAS**NG**GGKQALETVQRLLPVLCQAHGLTPDQVVAIAS**NN**GGKQALETVQRLLPVLC
QAHGLTPAQVVAIAS**HD**GGKQALETVQRLLPVLCQDHGLTPDQVVAIAS**NG**GGKQALETVQRLLPVL
CQAHGLTPDQVVAIAS**HD**GGKQALETVQRLLPVLCQAHGLTPDQVVAIAS**NI**GGKQALETVQRLLPVL
CQAHGLTPAQVVAIAS**HD**GGKQALETVQRLLPVLCQAHGLTPAQVVAIAS**HD**GGKQALETVQRLLPV
LCQDHGLTPAQVVAIAS**HD**GGKQALETVQRLLPVLCQAHG

ND4 Left TALE

NLTPDQVVAIAS**NN**GGKQALETVQRLLPVLCQAHGLTPAQVVAIAS**HD**GGKQALETVQRLLPVLCQD
HGLTPDQVVAIAS**NG**GGKQALETVQRLLPVLCQAHGLTPAQVVAIAS**NI**GGKQALETVQRLLPVLCQA
HGLTPEQVVAIAS**NN**GGKQALETVQRLLPVLCQAHGLTPDQVVAIAS**NG**GGKQALETVQRLLPVLCQ
DHGLTPDQVVAIAS**NI**GGKQALETVQRLLPVLCQAHGLTPDQVVAIAS**NI**GGKQALETVQRLLPVLCQ
AHGLTPAQVVAIAS**HD**GGKQALETVQRLLPVLCQAHGLTPDQVVAIAS**HD**GGKQALETVQRLLPVLC
QAHGLTPDQVVAIAS**NI**GGKQALETVQRLLPVLCQAHGLTPAQVVAIAS**HD**GGKQALETVQRLLPVLC
QAHGLTPDQVVAIAS**NN**GGKQALETVQRLLPVLCQAHGLTPDQVVAIAS**NG**GGKQALETVQRLLPVL
CQAHGLTPAQVVAIAS**NG**GGKQALETVQRLLPVLCQDHGLTPEQVVAIAS**HD**GGKQALETVQRLLPV
LCQAHG

ND4 Right TALE

NLTPDQVVAIAS**HD**GGKQALETVQRLLPVLCQAHGLTPAQVVAIAS**HD**GGKQALETVQRLLPVLCQD
HGLTPAQVVAIAS**NG**GGKQALETVQRLLPVLCQDHGLTPDQVVAIAS**NN**GGKQALETVQRLLPVLCQ
AHGLTPAQVVAIAS**NG**GGKQALETVQRLLPVLCQDHGLTPAQVVAIAS**NI**GGKQALETVQRLLPVLCQ
AHGLTPAQVVAIAS**NI**GGKQALETVQRLLPVLCQAHGLTPEQVVAIAS**NN**GGKQALETVQRLLPVLCQ
AHGLTPDQVVAIAS**NG**GGKQALETVQRLLPVLCQAHGLTPAQVVAIAS**NI**GGKQALETVQRLLPVLCQ
AHGLTPAQVVAIAS**NN**GGKQALETVQRLLPVLCQDHGLTPAQVVAIAS**NN**GGKQALETVQRLLPVLC
QAHGLTPDQVVAIAS**NI**GGKQALETVQRLLPVLCQDHGLTPAQVVAIAS**NN**GGKQALETVQRLLPVLC
QDHGLTPDQVVAIAS**NI**GGKQALETVQRLLPVLCQDHGLTPAQVVAIAS**NN**GGKQALETVQRLLPVLC
QDHG

ND5 Left TALE

NLTPAQVVAIAS**NI**GGKQALETVQRLLPVLCQDHGLTPDQVVAIAS**NN**GGKQALETVQRLLPVLCQDH
GLTPEQVVAIAS**HD**GGKQALETVQRLLPVLCQAHGLTPAQVVAIAS**NI**GGKQALETVQRLLPVLCQAH
GLTPDQVVAIAS**NG**GGKQALETVQRLLPVLCQAHGLTPDQVVAIAS**NG**GGKQALETVQRLLPVLCQA
HGLTPAQVVAIAS**NI**GGKQALETVQRLLPVLCQDHGLTPDQVVAIAS**NN**GGKQALETVQRLLPVLCQD
HGLTPEQVVAIAS**HD**GGKQALETVQRLLPVLCQAHGLTPAQVVAIAS**NI**GGKQALETVQRLLPVLCQA
HGLTPAQVVAIAS**NN**GGKQALETVQRLLPVLCQDHGLTPAQVVAIAS**NN**GGKQALETVQRLLPVLCQ

AHGLTPDQVVAIAS**NI**GGKQALETVQRLLPVLCQAHGLTPDQVVAIAS**NI**GGKQALETVQRLLPVLCQ
AHG

ND5 Right TALE

NLTPAQVVAIAS**NN**GGKQALETVQRLLPVLCQDHGLTPAQVVAIAS**NN**GGKQALETVQRLLPVLCQD
HGLTPDQVVAIAS**NG**GGKQALETVQRLLPVLCQAHGLTPAQVVAIAS**HD**GGKQALETVQRLLPVLCQ
AHGLTPDQVVAIAS**NG**GGKQALETVQRLLPVLCQAHGLTPAQVVAIAS**NG**GGKQALETVQRLLPVLC
QDHGLTPEQVVAIAS**NG**GGKQALETVQRLLPVLCQAHGLTPAQVVAIAS**NN**GGKQALETVQRLLPVL
CQDHGLTPDQVVAIAS**NN**GGKQALETVQRLLPVLCQAHGLTPAQVVAIAS**NI**GGKQALETVQRLLPVL
CQAHGLTPEQVVAIAS**NN**GGKQALETVQRLLPVLCQAHGLTPDQVVAIAS**NG**GGKQALETVQRLLPV
LCQDHGLTPDQVVAIAS**NI**GGKQALETVQRLLPVLCQDHGLTPAQVVAIAS**NN**GGKQALETVQRLLPV
LCQDHGLTPDQVVAIAS**NI**GGKQALETVQRLLPVLCQAHG

ND6 Left TALE

NLTPAQVVAIAS**HD**GGKQALETVQRLLPVLCQDHGLTPEQVVAIAS**NI**GGKQALETVQRLLPVLCQAH
GLTPAQVVAIAS**NI**GGKQALETVQRLLPVLCQDHGLTPAQVVAIAS**HD**GGKQALETVQRLLPVLCQAH
GLTPDQVVAIAS**HD**GGKQALETVQRLLPVLCQDHGLTPDQVVAIAS**HD**GGKQALETVQRLLPVLCQD
HGLTPAQVVAIAS**HD**GGKQALETVQRLLPVLCQAHGLTPAQVVAIAS**NG**GGKQALETVQRLLPVLCQ
DHGLTPDQVVAIAS**NN**GGKQALETVQRLLPVLCQAHGLTPEQVVAIAS**NI**GGKQALETVQRLLPVLCQ
AHGLTPAQVVAIAS**HD**GGKQALETVQRLLPVLCQAHGLTPAQVVAIAS**HD**GGKQALETVQRLLPVLC
QDHGLTPAQVVAIAS**HD**GGKQALETVQRLLPVLCQDHGLTPAQVVAIAS**HD**GGKQALETVQRLLPVL
CQAHGLTPAQVVAIAS**HD**GGKQALETVQRLLPVLCQDHGLTPDQVVAIAS**NI**GGKQALETVQRLLPVL
CQAHG

ND6 Right TALE

NLTPEQVVAIAS**NI**GGKQALETVQRLLPVLCQAHGLTPDQVVAIAS**HD**GGKQALETVQRLLPVLCQAH
GLTPDQVVAIAS**NG**GGKQALETVQRLLPVLCQDHGLTPDQVVAIAS**NI**GGKQALETVQRLLPVLCQD
HGLTPDQVVAIAS**HD**GGKQALETVQRLLPVLCQDHGLTPAQVVAIAS**NI**GGKQALETVQRLLPVLCQA
HGLTPAQVVAIAS**NN**GGKQALETVQRLLPVLCQAHGLTPDQVVAIAS**HD**GGKQALETVQRLLPVLCQ
DHGLTPAQVVAIAS**NN**GGKQALETVQRLLPVLCQAHGLTPEQVVAIAS**NI**GGKQALETVQRLLPVLCQ

AHGLTPAQVVAIAS**NG**GGKQALETVQRLLPVLCQAHGLTPAQVVAIAS**NN**GGKQALETVQRLLPVLC
QDHGLTPDQVVAIAS**NN**GGKQALETVQRLLPVLCQAHGLTPAQVVAIAS**HD**GGKQALETVQRLLPVL
CQDHGLTPDQVVAIAS**NG**GGKQALETVQRLLPVLCQAHGLTPEQVVAIAS**NI**GGKQALETVQRLLPVL
CQAHGLTPDQVVAIAS**NG**GGKQALETVQRLLPVLCQDHG

ATP8 Left TALE

NLTPAQVVAIAS**NI**GGKQALETVQRLLPVLCQAHGLTPDQVVAIAS**NG**GGKQALETVQRLLPVLCQAH
GLTPDQVVAIAS**NG**GGKQALETVQRLLPVLCQAHGLTPDQVVAIAS**NI**GGKQALETVQRLLPVLCQAH
GLTPAQVVAIAS**NI**GGKQALETVQRLLPVLCQDHGLTPAQVVAIAS**NI**GGKQALETVQRLLPVLCQAH
GLTPDQVVAIAS**HD**GGKQALETVQRLLPVLCQAHGLTPDQVVAIAS**NI**GGKQALETVQRLLPVLCQAH
GLTPAQVVAIAS**HD**GGKQALETVQRLLPVLCQAHGLTPDQVVAIAS**NI**GGKQALETVQRLLPVLCQAH
GLTPAQVVAIAS**NI**GGKQALETVQRLLPVLCQDHGLTPAQVVAIAS**NI**GGKQALETVQRLLPVLCQAH
GLTPDQVVAIAS**HD**GGKQALETVQRLLPVLCQAHGLTPAQVVAIAS**NG**GGKQALETVQRLLPVLCQD
HGLTPDQVVAIAS**NI**GGKQALETVQRLLPVLCQAHG

ATP8 Right TALE

NLTPAQVVAIAS**NI**GGKQALETVQRLLPVLCQAHGLTPDQVVAIAS**NG**GGKQALETVQRLLPVLCQAH
GLTPDQVVAIAS**NG**GGKQALETVQRLLPVLCQAHGLTPDQVVAIAS**NG**GGKQALETVQRLLPVLCQA
HGLTPAQVVAIAS**NG**GGKQALETVQRLLPVLCQAHGLTPEQVVAIAS**NG**GGKQALETVQRLLPVLCQ
AHGLTPEQVVAIAS**NI**GGKQALETVQRLLPVLCQAHGLTPAQVVAIAS**NG**GGKQALETVQRLLPVLCQ
AHGLTPAQVVAIAS**NN**GGKQALETVQRLLPVLCQAHGLTPEQVVAIAS**NN**GGKQALETVQRLLPVLC
QAHGLTPAQVVAIAS**NN**GGKQALETVQRLLPVLCQDHGLTPDQVVAIAS**HD**GGKQALETVQRLLPVL
CQDHGLTPDQVVAIAS**NG**GGKQALETVQRLLPVLCQAHGLTPAQVVAIAS**NG**GGKQALETVQRLLPV
LCQAHGLTPEQVVAIAS**NG**GGKQALETVQRLLPVLCQAHGLTPAQVVAIAS**NN**GGKQALETVQRLLP
VLCQAHGLTPAQVVAIAS**NN**GGKQALETVQRLLPVLCQAHG
