# Supplementary material for: Precision mitochondrial DNA editing with high-fidelity DddA-derived base editors
Source: Nat Biotechnol. 2022 Oct 13;41(3):378–86. doi: 10.1038/s41587-022-01486-w (PMC10017512; doi:10.1038/s41587-022-01486-w)
Supplement: Supplementary file 2 — Reporting Summary [file 41587_2022_1486_MOESM2_ESM.pdf]

Corresponding author(s): Jin-Soo Kim

Last updated by author(s): Aug 21, 2022

Reporting Summary

Nature Research wishes to improve the reproducibility of the work that we publish. This form provides structure for consistency and transparency in reporting. For further information on Nature Research policies, see our Editorial Policies and the Editorial Policy Checklist.

Statistics

For all statistical analyses, confirm that the following items are present in the figure legend, table legend, main text, or Methods section.

n/a	Confirmed	
☐	☒	The exact sample size (*n*) for each experimental group/condition, given as a discrete number and unit of measurement
☐	☒	A statement on whether measurements were taken from distinct samples or whether the same sample was measured repeatedly
☐	☒	The statistical test(s) used AND whether they are one- or two-sided<br>*Only common tests should be described solely by name; describe more complex techniques in the Methods section.*
☒	☐	A description of all covariates tested
☒	☐	A description of any assumptions or corrections, such as tests of normality and adjustment for multiple comparisons
☐	☒	A full description of the statistical parameters including central tendency (e.g. means) or other basic estimates (e.g. regression coefficient) AND variation (e.g. standard deviation) or associated estimates of uncertainty (e.g. confidence intervals)
☐	☒	For null hypothesis testing, the test statistic (e.g. *F*, *t*, *r*) with confidence intervals, effect sizes, degrees of freedom and *P* value noted<br>*Give P values as exact values whenever suitable.*
☒	☐	For Bayesian analysis, information on the choice of priors and Markov chain Monte Carlo settings
☒	☐	For hierarchical and complex designs, identification of the appropriate level for tests and full reporting of outcomes
☒	☐	Estimates of effect sizes (e.g. Cohen's *d*, Pearson's *r*), indicating how they were calculated

Our web collection on statistics for biologists contains articles on many of the points above.

Software and code

Policy information about availability of computer code

Data collection	For structural assay, we used PyMOL (2.5.3) and custom Python script described in Supporting Information.
Data analysis	High-throughput sequencing data was analyzed using CRISPR RGEN Tools (http://www.rgenome.net/) and Geneious (version 2022.0.1). Microsoft Excel (2019), Powerpoint (2019), and Prism 9 (9.3.1) was used for drawing figures, graphs, and tables. Genome alignment, primer design, and cloning design were performed with Geneious (version 2022.0.1) using NC_012920 as a reference. Further details and references and provided in the Methods.

For manuscripts utilizing custom algorithms or software that are central to the research but not yet described in published literature, software must be made available to editors and reviewers. We strongly encourage code deposition in a community repository (e.g. GitHub). See the Nature Research guidelines for submitting code & software for further information.

Data

Policy information about availability of data

All manuscripts must include a data availability statement. This statement should provide the following information, where applicable:

- Accession codes, unique identifiers, or web links for publicly available datasets
- A list of figures that have associated raw data
- A description of any restrictions on data availability

All data supporting the results are available in the main text or supplementary materials. The data that support the findings of this study are available from the corresponding author upon request. The high-throughput sequencing data from this study have been deposited in the NCBI Sequence Read Archive (SRA) database under the accession codes PRJNA817018 and PRJNA84738124. Python code used in this study is described in the supplementary materials.

Field-specific reporting

Please select the one below that is the best fit for your research. If you are not sure, read the appropriate sections before making your selection.

☒ Life sciences ☐ Behavioural & social sciences ☐ Ecological, evolutionary & environmental sciences

For a reference copy of the document with all sections, see nature.com/documents/nr-reporting-summary-flat.pdf

Life sciences study design

All studies must disclose on these points even when the disclosure is negative.

Sample size	No statistical methods were used to predetermine sample size because it doesn't apply to this study.
Data exclusions	There were no data exclusions.
Replication	All samples were evaluated in at least biological independent duplicates (n=2) or triplicates (n=3) and results were reliably reproduced under the conditions tested.
Randomization	Randomization was not required in this study, because we analyzed all experiments performed and it is not related to clinical samples.
Blinding	Sample collection and analysis were performed by different researchers, without any description.

Reporting for specific materials, systems and methods

We require information from authors about some types of materials, experimental systems and methods used in many studies. Here, indicate whether each material, system or method listed is relevant to your study. If you are not sure if a list item applies to your research, read the appropriate section before selecting a response.

Materials & experimental systems

n/a	Involved in the study	
☐	☒	Antibodies
☐	☒	Eukaryotic cell lines
☒	☐	Palaeontology and archaeology
☒	☐	Animals and other organisms
☒	☐	Human research participants
☒	☐	Clinical data
☒	☐	Dual use research of concern

Methods

n/a	Involved in the study	
☒	☐	ChIP-seq
☒	☐	Flow cytometry
☒	☐	MRI-based neuroimaging

Antibodies

Antibodies used	Anti-FLAG tag (SAB4301135, Sigma Aldrich), anti-HA tag (ab215069, abcam), b-actin (sc-47778), anti-mouse (sc-2005), anti-rabbit (sc-2004), anti-goat (sc-2020) (Santa Cruz). Detailed dilutions are described in the method section.
Validation	Anti-FLAG: validated by manufacturer by western blotting against whole cell lysates from HEK293T cell line.<br>Anti-HA: validated by manufacturer by western blotting HA-tagged Human Glutaminase C overexpression lysates.<br>Anti-b-actin: validated by manufacturer by western blotting against whole cell lysates from HeLa cell line.<br>Anti-mouse IgG-HRP: validated by manufacturer by western blotting against whole cell lysates from HeLa cell line.<br>Anti-rabbit IgG-HRP: validated by manufacturer by western blotting against whole cell lysates from HeLa cell line.<br>Anti-goat IgG-HRP: validated by manufacturer by western blotting against whole cell lysates from HeLa cell line.

Eukaryotic cell lines

Policy information about cell lines

Cell line source(s)	HEK293T/17 (CRL-11268, American Type Culture Collection).
Authentication	No cell lines were authenticated.
Mycoplasma contamination	Cells were not tested for mycoplasma contamination.
Commonly misidentified lines (See ICLAC register)	No commonly misidentified lines were used.
