# Supplementary figures and images for: Precision mitochondrial DNA editing with high-fidelity DddA-derived base editors
Source: Nat Biotechnol. 2022 Oct 13;41(3):378–86. doi: 10.1038/s41587-022-01486-w (PMC10017512; doi:10.1038/s41587-022-01486-w)

Uncropped image for Fig. S3

Left: HA tag

Right: FLAG tag

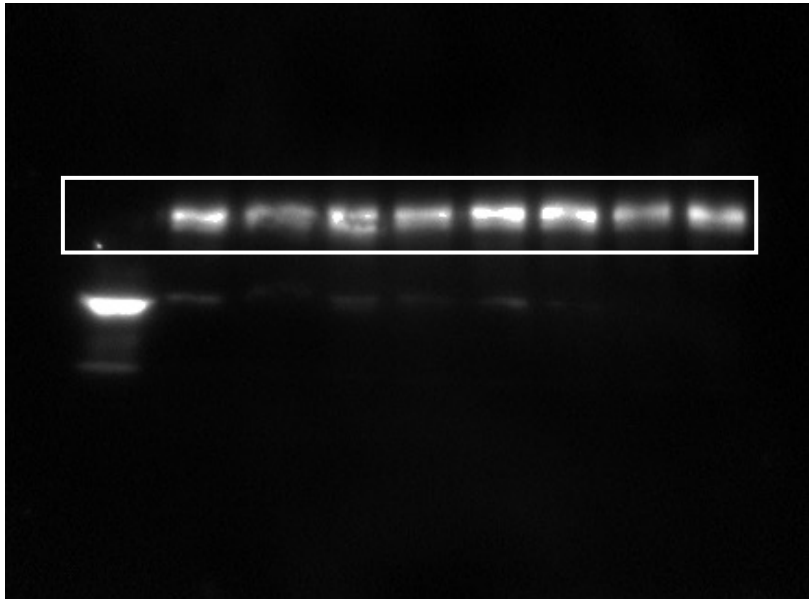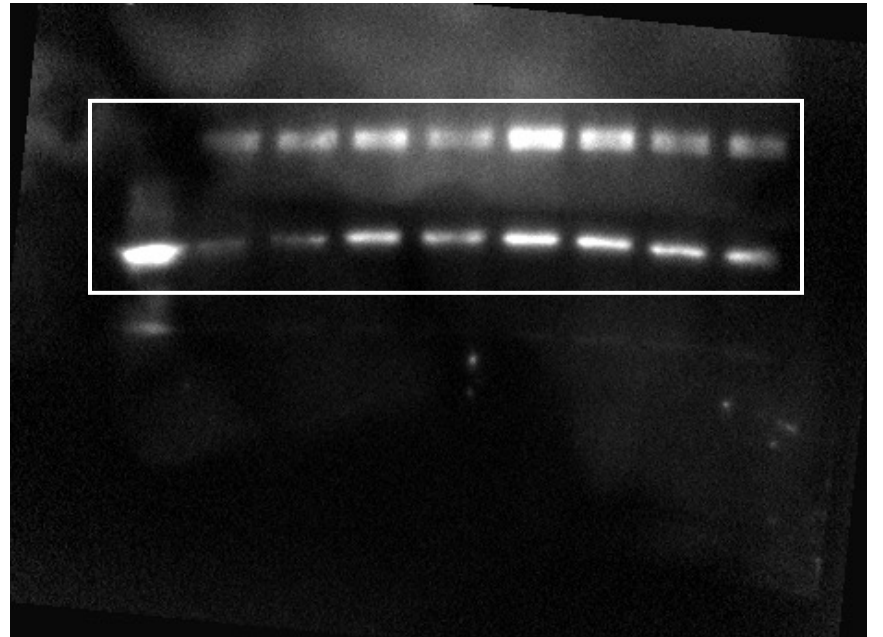

Supplement: Supplementary file 4 — Uncropped blot images for Supplementary figure S3. [file 41587_2022_1486_MOESM4_ESM.pdf]
